# Designed architectural proteins that tune DNA looping in bacteria

**DOI:** 10.1093/nar/gkab759

**Published:** 2021-09-03

**Authors:** David H Tse, Nicole A Becker, Robert T Young, Wilma K Olson, Justin P Peters, Tanya L Schwab, Karl J Clark, L James Maher

**Affiliations:** Department of Biochemistry and Molecular Biology, Mayo Clinic College of Medicine and Science, 200 First St. SW, Rochester, MN 55905, USA; Department of Biochemistry and Molecular Biology, Mayo Clinic College of Medicine and Science, 200 First St. SW, Rochester, MN 55905, USA; Department of Chemistry and Chemical Biology, Rutgers, the State University of New Jersey, Center for Quantitative Biology, Piscataway, NJ 08854, USA; Department of Chemistry and Chemical Biology, Rutgers, the State University of New Jersey, Center for Quantitative Biology, Piscataway, NJ 08854, USA; Department of Chemistry and Biochemistry, University of Northern Iowa, 1227 West 27th Street, Cedar Falls, IA 50614, USA; Department of Biochemistry and Molecular Biology, Mayo Clinic College of Medicine and Science, 200 First St. SW, Rochester, MN 55905, USA; Department of Biochemistry and Molecular Biology, Mayo Clinic College of Medicine and Science, 200 First St. SW, Rochester, MN 55905, USA; Department of Biochemistry and Molecular Biology, Mayo Clinic College of Medicine and Science, 200 First St. SW, Rochester, MN 55905, USA

## Abstract

Architectural proteins alter the shape of DNA. Some distort the double helix by introducing sharp kinks. This can serve to relieve strain in tightly-bent DNA structures. Here, we design and test artificial architectural proteins based on a sequence-specific Transcription Activator-like Effector (TALE) protein, either alone or fused to a eukaryotic high mobility group B (HMGB) DNA-bending domain. We hypothesized that TALE protein binding would stiffen DNA to bending and twisting, acting as an architectural protein that antagonizes the formation of small DNA loops. In contrast, fusion to an HMGB domain was hypothesized to generate a targeted DNA-bending architectural protein that facilitates DNA looping. We provide evidence from *Escherichia coli* Lac repressor gene regulatory loops supporting these hypotheses in living bacteria. Both data fitting to a thermodynamic DNA looping model and sophisticated molecular modeling support the interpretation of these results. We find that TALE protein binding inhibits looping by stiffening DNA to bending and twisting, while the Nhp6A domain enhances looping by bending DNA without introducing twisting flexibility. Our work illustrates artificial approaches to sculpt DNA geometry with functional consequences. Similar approaches may be applicable to tune the stability of small DNA loops in eukaryotes.

## Graphical Abstract

**Graphical Abstract:**
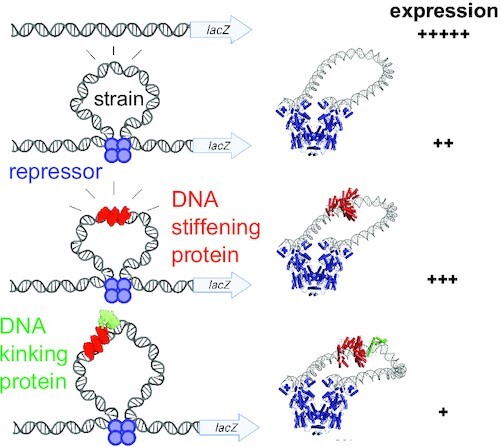
The stability of Lac repressor loops in *E. coli* is tuned by designed artificial sequence-specific architectural DNA binding proteins that stiffen or kink looped DNA, impacting gene expression in opposite ways.

## INTRODUCTION

Duplex DNA contains the genetic information of cells. DNA is also one of the stiffest biopolymers (1). Overcoming DNA stiffness is important for various biological processes such as compaction of viral DNA into bacteriophage heads (2) and formation of small DNA loops (3). The importance of DNA looping cannot be overstated. Distant sites separated across the genome are brought together in transcriptional gene regulation and recombination (4,5). In some cases, multiple genes are transcribed together through shared RNA polymerases in a transcription ‘factory’ (6). Smaller DNA loops between sites separated by few turns of the stiff DNA double helix are formed in the case of Lac repressor or phage λ repressor (7,8). Control of these processes might be achieved through the artificial manipulation of DNA stiffness. Here, we explore the design and testing of sequence-specific architectural DNA binding proteins to tune DNA looping over short distances in bacteria where resistance of DNA to bending and twisting is dominant. We achieve this by engineering Transcription Activator-like Effector (TALE) proteins with or without fusion to a eukaryotic high mobility group B (HMGB) domain capable of inducing sharp DNA bending. Part of this work emulates designed proteins features of tight DNA loop enhancement by natural sequence-nonspecific architectural proteins (9,10). Other groups have previously engineered transcription factors or Cas9 to regulate DNA looping (11,12). In addition, prior work from our laboratory has shown evidence that free yeast Nhp6A architectural protein can bind within strained Lac repressor loops in engineered bacteria (10). The present work is novel in targeting the sequence-nonspecific Nhp6A protein by fusion to a sequence-specific TALE protein.

TALEs were discovered as virulence factors in phytopathogenic bacteria from the genus *Xanthomonas*. These bacteria utilize a type III secretion system to inject TALEs into plant cells to activate genes that apparently facilitate infection (13). TALEs bind DNA in a remarkable sequence-specific manner through well-characterized repeats that consist of 33–35 amino acid modules differing only at positions 12 and 13 (the repeat variable diresidue, RVD). Each diresidue determines the base pair recognition specificity of each corresponding TALE module (14). Crystal structures of TALE binding to DNA show the direct contact between the side chain of residue 13 and the target DNA base, thereby contributing to DNA-binding specificity (15,16). TALEs found in nature range from 2 to 34 repeats, with the majority being 16–20 repeats in length (13). For engineered TALEs, 16–25 repeats correspond to optimum target sequence specificity with reduced non-target binding, consistent with the median length of naturally-occurring TALEs (17).

While no prior studies have addressed the impact of TALE binding on DNA flexibility, inspection of X-ray crystal structures suggests that the TALE amino acids fully engage the DNA major groove (15,16). We hypothesized that this engagement would limit DNA bending and twisting motions, effectively stiffening the DNA polymer. Such stiffening could have the effect of increasing the energy of DNA looping by constraining DNA bending and twisting to a smaller number of base pairs within the loop. In this way, TALEs might serve as artificial architectural DNA binding proteins that tune DNA looping by making it more expensive.

Eukaryotic high mobility group (HMG) non-histone proteins were first isolated almost fifty years ago and named for their electrophoretic mobility in denaturing polyacrylamide gels (18). Many such proteins bind to DNA in a sequence-nonspecific manner. HMG proteins are subdivided into three superfamilies (19), HMGB, HMGN, and HMGA. These proteins act as accessory architectural factors involved in nucleosome and chromatin modulation (20). Architectural proteins are also believed to regulate other nuclear activities including transcription, replication, and DNA repair (21–25). Some HMGB proteins contain two tandem HMG box domains preceded or followed by protein segments rich in acidic residues. Each HMG box is composed of three α-helices that fold into an L-shaped conformation and can bind to the minor groove of DNA, accompanied by intercalation of certain side chains with low sequence specificity (26). Introduction of the hydrophobic surface of the HMG box domain into the minor groove of DNA causes an approximately 90° kink (27). Transient sharp bending of DNA at random positions amounts to an enhancement in DNA chain flexibility (28). Nhp6A is a single-box HMGB protein from *Saccharomyces cerevisiae* (29). This sequence-nonspecific architectural protein bears structural similarities to each of the two HMG boxes of mammalian HMGB1/2 proteins. In the present study, Nhp6A is fused to a sequence-specific DNA-binding protein to create a novel architectural DNA binding protein that targets a DNA kink to a desired site within a DNA repression loop. It remains unknown whether the strong bend induced by Nhp6A has flexible hinge character (30,31).

TALEs have been shown to be effective for tethering other functions such as endonucleases (32–34). We therefore hypothesized that TALE-HMGB protein fusions might serve as artificial sequence-specific architectural proteins that deliver sharp DNA bends, facilitating DNA looping. Such designed artificial architectural DNA binding proteins would offer new tools for programing the three-dimensional structure of DNA. We set about to test these ideas in this work.

**Figure 1.**
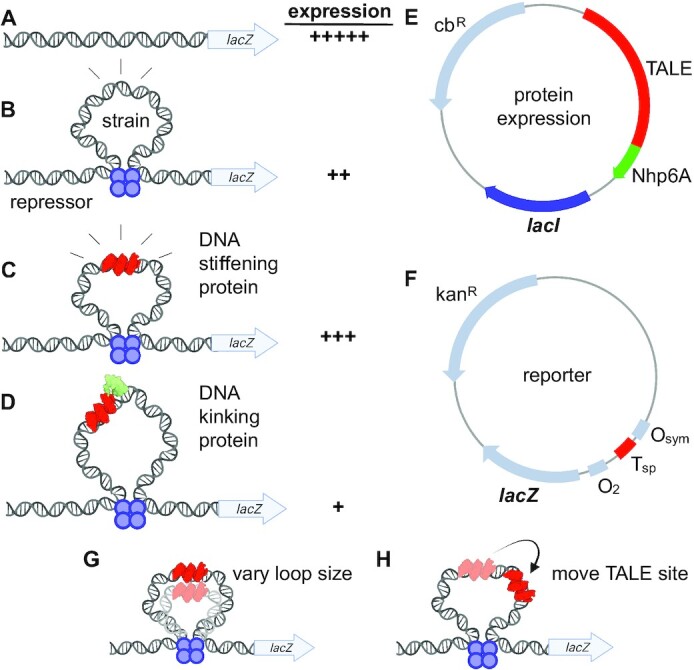
Concepts addressed in this work. (**A**) Reporter construct in the absence of Lac repressor-mediated looping is predicted to be strongly expressed. (**B**) Small, strained DNA loops anchored by Lac repressor should substantially repress the promoter of the reporter construct. (**C**) It is hypothesized that a TALE protein targeted to the Lac repressor loop will serve as an artificial architectural DNA binding protein that stiffens the bound DNA segment to bending and twisting, limiting deformations to unbound segments of the loop, and thereby shifting the equilibrium away from looping and causing derepression. (**D**) It is hypothesized that endowing the site-specific TALE protein with a sequence-nonspecific Nhp6A DNA-kinking domain will relieve bending strain, improving repression by Lac repressor-mediated looping. (**E**) Schematic illustration of protein expression plasmid providing TALE protein with or without Nhp6A fusion, and/or Lac repressor. (**F**) Schematic illustration of experimental reporter construct to evaluate detailed effects of artificial sequence-specific architectural proteins. (**G**) Scheme for reporter constructs that vary loop size with a fixed TALE position. (**H**) Scheme for reporter constructs that vary TALE position for a fixed loop size.

Our approach is summarized in Figure 1. We hypothesized that a strongly-expressed reporter gene (Figure 1A) will be repressed by a small and strained DNA loop (Figure 1B), and that this repression can be tuned by artificial TALE-based architectural DNA binding proteins that either stiffen the looped DNA leading to derepression (Figure 1C), or that introduce a kink within the loop, facilitating repression (Figure 1D). Lac repressor and TALE derivatives (Figure 1E) are expressed on a separate plasmid in addition to a *lacZ*-based reporter plasmid (Figure 1F). The impacts of designed artificial architectural DNA binding proteins were assessed in sets of experiments where the architectural protein was placed at a fixed position relative to the downstream operator as loop size was systematically changed (Figure 1G), or systematically moved within a DNA loop of fixed length (Figure 1H).

## MATERIALS AND METHODS

### DNA looping reporter constructs

DNA looping reporter constructs were based on plasmid pJ2656, a derivative of pJ2490 containing an upstream multiple cloning site region (35), containing the *lac* uv5 promoter and a single proximal O_2_ operator. Molecular cloning was used to install a 15-bp specific TALE recognition sequence (T_sp_) (Supplementary Table S1) – top strand: tTCATGTTATAACGGA to generate plasmid pJ2721 containing the T_sp_ site upstream of the proximal O_2_ operator as the basis for Series 1 constructs. The 5′ invariant thymine residue of the TALE target is shown in lower case. Various oligonucleotide duplexes containing the O_sym_ operator were then cloned to generate pJ2721-pJ2737 constructs (Supplementary Table S2; Figure S1) with spacings of 131.5–146.5-bp (measured center-to-center between O_2_ and O_sym_ operators). To facilitate cloning of Series 2 constructs, plasmid pJ2748 was created by adding a restriction endonuclease recognition site to pJ2721. Plasmid pJ2748 served as the basis of the cloning of Series 2 constructs. Duplexes adjust the position of the T_sp_ recognition sequence in base pair increments in the context of a constant 142.5-bp DNA loop length (Supplementary Table S3; Figure S2). All looping reporter constructs contain a p15A, low copy number origin of replication, and are from a different compatibility group than plasmids expressing TALE DNA binding proteins to allow for co-transformation (36).

### Expression of DNA binding proteins

**Figure 2.**
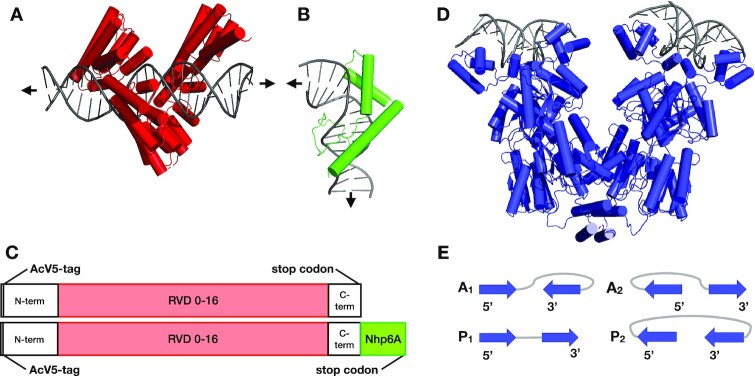
Engineered fusion proteins and the Lac repressor. (**A**) General TALE structure (red) bound to DNA (grey) based on PDB: 3UGM, but with 15-bp target site as in this work. The depicted structure (red) includes only the 15 repeated DNA recognition domains. N- and C-terminal domains are not shown. For additional sequence information, see Supplementary Figure S3. (**B**) Full Nhp6A structure (green) bound to DNA (grey) based on PDB: 1CG7. Arrows indicate DNA trajectory. (**C**) Domain structure of fusion proteins drawn approximately to scale. (**D**) Structure of the repressor (blue) in complex with operator DNA (grey) based on PDB: 1EFA, 1LBI. (**E**) Schematics of repressor-mediated DNA looping, with arrows depicting the 5′-3′ directions of operators on the binding headpieces, characters A/P specifying the antiparallel or parallel orientations of bound operators, and numerals 1/2 distinguishing whether the first operator (O_sym_) points toward the inside or outside of the assembly.

TALE expression plasmids, pJ2652 and pJ2654, are based on plasmid TALE-FKBP F36M (36) and contain the pMB1, low copy number origin of replication (37). These TALE proteins contain an N-terminal AcV5 epitope tag to allow for expression monitoring by Western blotting (Figure 2A–C). The TALE repeat region was inserted as described (38). Plasmid pJ2652 encodes the specific TALE (sp TALE) protein recognizing the T_sp_ sequence. Plasmid pJ2654 encodes a different TALE (ns TALE) protein recognizing a different sequence (T_ns_ – top strand: tTACAAGTGGCTCATT) (Supplementary Tables S1 and S4) (36). DNA encoding the yeast Nhp6A protein was amplified from plasmid J2472 and this coding sequence was used create TALE-Nhp6A fusions (Supplementary Table S4; Figure S3). The *lacI* gene encoding Lac repressor was amplified from plasmid pJ2179 and installed downstream from the TALE coding sequence in some cases.

Protein expression plasmids were transformed into electrocompetent bacterial strain FW102 [*araD(gpt-lac)5(Str^R^*)] (39) and transformants selected on LB agar plates containing streptomycin and carbenicillin. The resulting bacteria were made electrocompetent and transformed with appropriate reporter plasmids with selection on LB agar plates containing streptomycin, carbenicillin and kanamycin.

### Western blotting

Bacterial strains were grown in 10 ml LB medium containing appropriate antibiotics overnight at 37°C with aeration. Saturated overnight culture (200 μl) was used to seed a 20-ml subculture on the next day and grown for 2–3 h at 37°C with aeration. When the OD_600_ of the subculture reached 0.4–0.6, 6 ml of the culture was pelleted by centrifugation and resuspended in 200 μl 1× NuPAGE MES SDS running buffer (Thermo). Cells were lysed by sonication and clarified by centrifugation. Protein was quantified using a BCA protein assay kit (Thermo). Protein (6 μg) was subjected to electrophoresis through a NuPAGE 10% Bis–Tris precast gels (Thermo) at 125 V for 2 h. Gels were then blotted onto PVDF membranes (Bio-Rad) using an XCell II Blot Module (Thermo) in 1 × NuPAGE transfer buffer (Thermo) containing 20% methanol. Blotted membrane was briefly washed in TBST (50 mM Tris–HCl, pH 7.4, 150 mM NaCl, 0.1% Tween 20), treated with blocking buffer (5% dry milk and 1% BSA in TBST) for 1 h at room temperature, incubated with primary antibody overnight at 4°C in TBST with gentle rocking. Membranes were washed with TBST three times for 10 min at room temperature and then incubated with secondary antibody in blocking buffer for 1 h at room temperature with gentle rocking. Membranes were analyzed with an Amersham Typhoon laser-scanner platform (Cytiva) using IRshort and IRlong methods. Uncropped images are shown in Supplementary Figure S4.

Primary antibodies for western blotting were as follows: mouse anti-AcV5 (1:2000, ab49581, abcam); rabbit anti-*E. coli* Lac repressor (1:2000, C60143, LSBio). Secondary antibodies for western blotting were as follows: IRDye 800CW goat anti-mouse IgG (1:10 000, 926–32210, Li-cor); IRDye 680LT goat anti-rabbit IgG (1:10 000, 926-68021, Li-cor).

### *E. coli* β-galactosidase reporter assay

Quantitation of DNA looping was accomplished by measurement of β-galactosidase activity in bacterial extracts as previously described (40). To establish ideal levels of Lac repressor activity, bacterial extracts were assayed after cell growth with or without 100 μM IPTG. β-Galactosidase reporter activity is presented in Miller units (MU). Repression level (RL) is calculated according to Equation (1):

(1) $$\begin{equation*}{\rm{RL}} = \frac{{{\rm{M}}{{\rm{U}}_{{\rm{TALE}},{\rm{\;}}{{\rm{O}}_2}, - {\rm{lacI}}}}}}{{{\rm{M}}{{\rm{U}}_{{\rm{TALE,}}{{\rm{O}}_{{\rm{sym}}}}{{\rm{O}}_{\rm{2}}}{\rm{, + lacI}}}}}}\;\end{equation*}$$

where TALE indicates the presence of any appropriate TALE protein. We note that prior experiments have shown that an isolated distal O_sym_ operator, with or without bound lacI, does not influence promoter activity in the absence of a proximal O_2_ operator. The normalized repression level (RL_n_) is given by Equation (2):

(2) $$\begin{equation*}{{\rm RL}_n} = \frac{{{{\rm RL}_{{\rm{sp\;TALE}}}}}}{{{{\rm RL}_{{\rm{ns\;TALE}}\;}}}}\;\end{equation*}$$

where sp TALE indicates the presence of the sp TALE protein with the target T_sp_ sequence in the reporter constructs, and ns TALE indicates the presence of the ns TALE protein with no binding site present in the reporter system. Data are provided in Supplementary Tables S5 and S6 with error bars representing the standard deviation of data from a minimum of six colonies for each data point.

Using a transformation of the data previously described (41), the repression level data can also be expressed in terms of a looping *J*-factor for each operator spacing (sp) according to Equation (3):

(3) $$\begin{equation*}{J_{{\rm{loop}}}}\left( {{\rm sp}} \right) = \frac{{\left( {{{\rm RL}_{{\rm{loop}}}}\left( {{\rm sp}} \right) - {{\rm RL}_{{\rm{noloop}}}}} \right)\left[ {{\rm{lacI}}} \right]}}{{{{\rm RL}_{{\rm{noloop}}}} - 1}}\end{equation*}$$

where RL_noloop_ is the repression level for the construct with only the proximal O_2_ operator and [lacI] is the cellular concentration of Lac repressor. We estimate the concentration of Lac repressor to be 100 nM based on (42).

### Data fitting to thermodynamic DNA looping model

Curve fitting to a thermodynamic model of *lac* promoter repression was performed using non-linear least-squares refinement to each set of RL data with five adjustable parameters for the variable O_sym_–O_2_ series (Series 1) and three adjustable parameters for the variable TALE–O_2_ series (Series 2), as described below (and see Table 1). The thermodynamic model that relates gene expression and spacing of operator sequences has been previously described (37,40,43). Fit parameters give insight into the physical properties of the nucleoprotein loops.

**Table 1.** Thermodynamic model fits for data

Parameter	sp TALE	sp TALE-Nhp6A	ns TALE	ns TALE-Nhp6A
	**O_**sym**_–O_**2**_ spacings**			
*hr* (bp/turn)	10.96 ± 0.04	10.98 ± 0.06	11.07 ± 0.02	11.13 ± 0.05
*C*_app_ (× 10^–19^ erg cm)	2.86 ± 0.07	2.11 ± 0.06	0.44 ± 0.04	0.31 ± 0.02
*K*_max_	9.82 ± 0.13	55.24 ± 0.98	33.56 ± 4.85	29.88 ± 5.11
*K*_NSL_	4.83 ± 0.03	11.48 ± 0.24	0 ± 5.11	0 ± 5.87
*sp*_optimal_ (bp)	142.65 ± 0.02	142.82 ± 0.03	144.03 ± 0.08	143.14 ± 0.24
	**TALE – O_2_ spacings**			
^TALE^ *C*_app_ (× 10^–19^ erg cm)	0.34 ± 0.01	0.36 ± 0.01	0.25 ± 0.01	0.24 ± 0.01
^TALE^ *K*_Smax_	15.62 ± 0.12	49.66 ± 0.97	42.03 ± 1.03	38.61 ± 0.99
^TALE^ *sp*_optimal_ (bp)	97.89 ± 0.04	94.79 ± 0.08	92.95 ± 0.15	93.18 ± 0.17

All parameters are + LacI in the presence of 100 μM IPTG and are presented with a 95% confidence interval.

The thermodynamic model is based on the premise that promoter repression is sensitive only to the occupancy of the downstream *lac* O_2_ operator at equilibrium (44–46). The extent of promoter repression is modeled by evaluating the distribution of possible states of this operator. If a singly-bound repressor exists at O_2_ (‘single bound’) driven only by the concentration of free repressor in the cell, operator occupancy may be modest and repression low. In contrast, repressor bound to the strong upstream O_sym_ operator increases local repressor concentration at the proximal operator (‘specific looping’) to drive more complete promoter repression through the mechanisms in question here.

In the variable O_sym_–O_2_ reporter constructs (Series 1), the fraction of proximal operator bound by repressor as a function of DNA operator–operator length is modeled with five adjustable parameters evaluating the distribution of possible states of the proximal operator through a partition function for the system (37,40,47,48). In this context, *hr* is the DNA helical repeat, *C*_app_ is the apparent torsional modulus of the DNA loop, *sp*_optimal_ is the optimal spacing between operators (in base pairs) where *sp* is the actual spacing for a given construct, *K*_max_ is the equilibrium constant for the formation of a specific loop with optimal phasing, and *K*_NSL_ is the equilibrium constant for all forms of O_sym_-dependent enhanced binding to O_2_ other than the specific loop.

In the variable TALE–O_2_ reporter constructs (Series 2), the distance between the downstream and upstream operators is held constant at 142.5 bp. The face of the helix occupied by various sp TALE proteins is then systematically changed in a manner that potentially influences loop stability: if TALE binding increases DNA strain, the loop is destabilized by decreased anchoring from repressor. If TALE binding decreases DNA strain, the loop is stabilized (‘stabilized loop’) and the repressed state is favored. Here, there are three adjustable parameters, since *hr* remains unchanged from fitting in the previous series. ^TALE^*C*_app_ is the apparent torsional modulus of the DNA loop, ^TALE^*sp*_optimal_ is the optimal spacing between the TALE binding site and O_2_ (in base pairs), and ^TALE^*K*_Smax_ is the equilibrium constant for the formation of a stabilized loop. The thermodynamic model that describes gene expression in the context where the loop size was held constant has been described (49).

### Molecular modeling

The configurations of DNA chains capable of looping between the headpieces of the Lac repressor assembly were obtained using a procedure that optimizes the energy of a collection of base pairs, in which the first and last pairs are held fixed (50). The DNA is described at the level of base-pair steps using six rigid-body parameters to specify the arrangements of successive base pairs—three angles (tilt, roll, twist) describing the orientation of successive base-pair planes and three translational components (shift, slide, rise) along the vector joining successive base-pair centers (51–53). The base pairs in contact with the repressor and TALE-Nhp6A constructs are held fixed, with rigid-body parameters assigned values extracted from high-resolution structures (16,29,54–57) (see Supporting Information). The protein-free steps are subject to a potential that allows for elastic deformations of DNA from its equilibrium structure (58). The steps are assigned the elastic properties of an ideal, inextensible, naturally straight double helix, with bending deformations consistent with the persistence length of mixed-sequence DNA (59), fluctuations in twist compatible with the topological properties of DNA minicircles (60,61), and a variable helical repeat. These features are expressed at the base-pair level in terms of an equilibrium rest state with null values of all rigid-body parameters other than twist and rise and a set of elastic constants impeding deviations of parameters. The twist is assigned a reference value of 360°/*n*, where *n* is the assumed number of base pairs per turn, and the rise a value of 3.4 Å, the distance between the planes of successive base pairs. The energy is increased by $\frac{1}{2}$*k*_B_*T* by a change in twist of 4.1°, a bending deformation of 4.8°, or a translational move of 0.02 Å, where *k*_B_ is the Boltzmann constant and *T* the absolute temperature. Stabilizing interactions between protein and DNA are not considered. Configurations with steric overlaps are discarded.

In the absence of knowledge of the directions in which the DNA operators attach to the arms of the repressor, each operator is placed in two orientations on the protein-binding headpieces, yielding four distinct loops (Figure 2DE)—two termed A_1_ and A_2_ with the 5′-3′ directions of the bound fragments running in nearly opposing (antiparallel) directions and two termed P_1_ and P_2_ with the fragments running in the same (parallel) direction (62). Moreover, each of these loops includes configurations from two competing topological families, with similar, albeit out-of-phase, dependencies on chain length (63,64), leading to eight potential spatial forms ${\rm{A}}_1^{{\rm{F1}}}{\rm{,A}}_1^{{\rm{F2}}},{\rm{A}}_2^{{\rm{F1}}}$, etc. distinguished by the family (F1, F2) and connectivity (1,2) of the loop. The leading strand of the O_sym_ operator at the start of the loop progresses toward the central axis of the protein assembly in configurations with a connectivity of 1 and toward the outside in those with a connectivity of 2.

Optimized structures of DNA loops of increasing chain length are obtained from the configurations of previously determined protein-free loops with 92-bp center-to-center operator spacing (64). Base pairs are added one at a time by assigning the coordinates of an arbitrary base pair in an existing structure to a new residue and then minimizing the energy of the enlarged system under the same end-to-end constraints. Proteins are introduced with a ramping procedure that gradually changes the rigid-body parameters of base-pair steps at the desired binding site from those of deformable, protein-free DNA. Each ramping step is accompanied by an optimization, which freezes the partially deformed protein-bound segment while reconfiguring the DNA loop.

Looping propensities, or *J*-factors, are estimated from the sums of the statistical weights, that is, the Boltzmann factors, of the eight energy-optimized configurations of a loop of given chain length in the presence or absence of a specifically bound TALE construct. This treatment ignores other features of the system that might contribute to the energy, for example, deformation of headpiece-bound DNA sufficient to wrap the loop around the repressor (65,66) or loss of intermolecular contacts that perturb the ends of operator DNA (56) and enhance the computed looping propensities (63). If a DNA loop is short enough, the lowest energy states mirror the looping propensities and modes of chain attachment captured in direct simulations of linear chain molecules subject to the same spatial constraints (63,67). The preferred modes of looping are measured in terms of the fractional contributions *f_i_* of each specific loop type *i* to the *J*-factor, that is,

(4) $$\begin{equation*}{f_i} = \frac{{{e^{{{ - {\psi _i}} \mathord{\left/ {\vphantom {{ - {\psi _i}} {{k_B}T}}} \right. } {{k_B}T}}}}}}{J},\end{equation*}$$

where Ψ*_i_* is the total elastic energy of the optimized loop and *J* is evaluated over the eight looped states.

(5) $$\begin{equation*}J = \sum\limits_{i = 1}^8 {e^{{ - {{\rm{\psi }}_1}} \mathord{\left/ {\vphantom {{ - {{\rm{\psi }}_1}} {{K_B}T}}} \right. } {{k_B}T}}.} \end{equation*}$$

## RESULTS AND DISCUSSION

### TALE-based architectural protein design

TALE and TALE-Nhp6A were created by replacing the FKBP (F36M) in TALE dimer constructs (36) with a stop codon or Nhp6A domain (Figure 2C; Supplementary Figure S3; Supplementary Table S4). N- and C-terminal TALE sequences are from natural TALE proteins and are contained within the parental vector with the central repeat domain added using the FusX assembly system (38). The source TALE structure for Figure 2A is PDB: 3UGM, which shows the TALE PthXo1 bound to its DNA target (16). The depicted structure shows only the 15 repeated DNA recognition domains. The source structure for Nhp6A shown in Figure 2B is 1CG7 (68) depicting the sequence-nonspecific Nhp6A-DNA complex with DNA strongly bent by two minor groove amino acid side chain intercalations and neutralizing cationic amino acids in the compressed major groove. The *lac* operon looping system is anchored through the binding of Lac repressor tetramer at O_sym_ and O_2_ operators (Figure 1B and 1F). The *lacI* gene encoding Lac repressor was cloned downstream of the desired TALE coding sequence. In this way, DNA looping was enabled or disabled by including or excluding Lac repressor from the plasmid.

### Characterization of components

**Figure 3.**
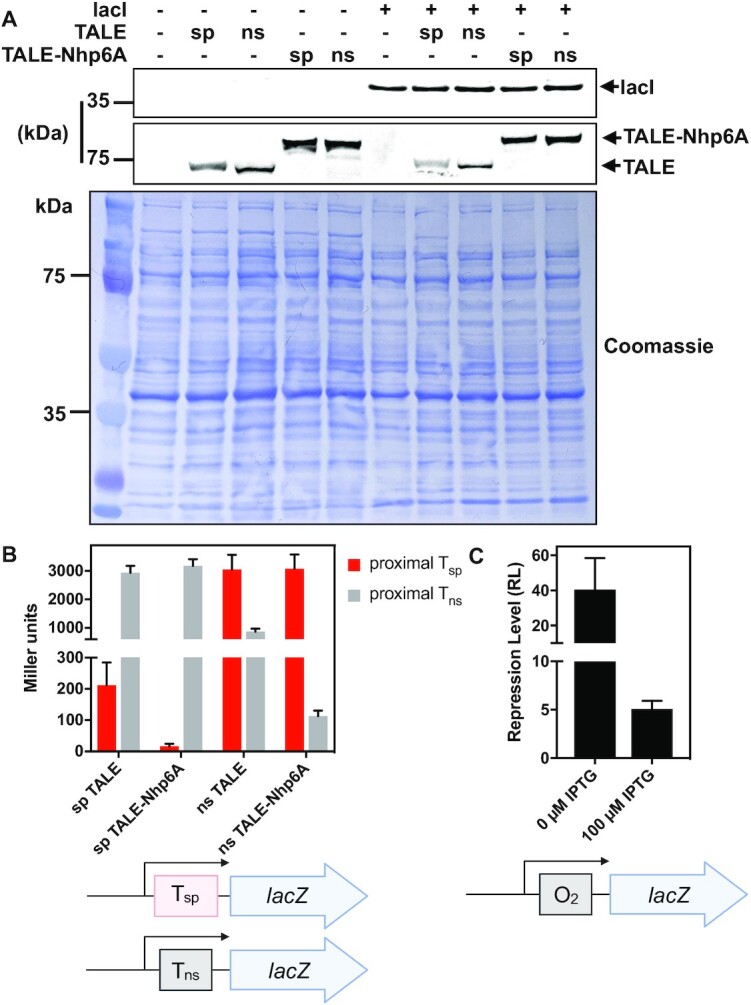
Lac repressor and TALE protein expression, TALE binding affinity and specificity, and Lac repressor tuning with IPTG. (**A**) Western blot analysis of lysates from *E. coli* expressing the indicated genes encoding specific (sp) or nonspecific (ns) TALE (75.4 kDa), or TALE-Nhp6A (86.2 kDa) fusion proteins with or without Lac repressor (38.6 kDa). TALE and TALE-Nhp6A fusion proteins contain an N-terminal AcV5 epitope. The sp and ns TALE constructs contain the same number of amino acids. The two constructs are identical in size. We interpret the subtle difference in SDS gel mobility as being due to small changes in SDS detergent interactions with the constructs based on subtle amino acid differences in the DNA recognition repeats. (**B**) *lacZ* expression when the indicated TALE (x-axis) is expressed with the indicated TALE binding sequence at the proximal operator position. (**C**) Desired tuning of effective Lac repressor affinity for test reporter construct with O_2_ in the proximal position.

TALE and TALE-Nhp6A were expressed in bacterial strain FW102 (39). Western blotting (Figure 3A) reveals that TALE, TALE-Nhp6A, and Lac repressor proteins are properly expressed at their expected molecular weights. Importantly, expressed levels of TALE proteins and Lac repressor are independent of each other. The DNA binding specificities and affinities of TALE proteins and fusions were assessed using β-galactosidase reporter assay (Figure 3B) where reporter constructs contained T_sp_ or T_ns_ DNA recognition sequences (Supplementary Table S1) in a position corresponding to the proximal operator but in the absence of Lac repressor. Binding of the TALE protein in this position inhibits transcription and subsequent *lacZ* expression. β-galactosidase reporter assays sensitively reflect TALE binding affinity, revealing high *lacZ* expression when sp TALE and sp TALE-Nhp6A are expressed with reporter constructs containing the non-cognate proximal TALE recognition sequence (Figure 3B). Conversely, *lacZ* expression is repressed when sp TALE and sp TALE-Nhp6A are co-expressed with a reporter plasmid containing a cognate proximal recognition sequence (Figure 3B). Likewise, co-expression of ns TALE and ns TALE-Nhp6A with a reporter construct containing a proximal T_ns_ DNA recognition sequence results in lower *lacZ* expression, though repression is not as strong as for sp TALE and its cognate T_sp_ site (Figure 3B, grey vs. red bars). Interestingly, binding of sp TALE-Nhp6A fusions causes greater repression than the corresponding TALE proteins (Figure 3B). This could imply a repressive effect of an induced DNA kink at the position of the Nhp6A module, or increased steric occlusion of the promoter by this C-terminal module. Other explanations include an increase in occupancy of the promoter by TALE-Nhp6A due to increased overall affinity through the addition of the sequence-nonspecific Nhp6A domain, or the slightly higher level of TALE-Nhp6A protein suggested by Figure 3A. Together, these data show that TALE constructs have high binding specificity for their target recognition sequences.

For convenience, the present assay system employs multi-copy plasmids rather than single-copy episomal constructs as we often study. This results in a higher Lac repressor expression and corresponding greater basal repression from reference reporters carrying a single weak pseudo-palindromic O_2_ operator in the proximal position [∼40-fold repression rather than ∼4-fold observed for Lac repressor expressed from a single-copy episome (37)]. To compensate and facilitate comparison with this earlier work, Lac repressor affinity was moderated by performing all experiments in the presence of a low concentration (100 μM) of IPTG inducer, bringing RL to ∼5 (Figure 3C). It is important to recognize that these studies therefore are exploring the interaction of designed architectural DNA binding proteins with DNA loops driven by populations of Lac repressor molecules with a low degree of IPTG saturation. It has been shown that IPTG-bound Lac repressor retains some affinity for *lac* operators, but displays altered properties relative to free Lac repressor (37). For the interested reader, similar experiments were also performed in the absence of IPTG, with similar results (Supplementary Figures S5 and S6 and Table S7).

### *lac* looping model system

**Figure 4.**
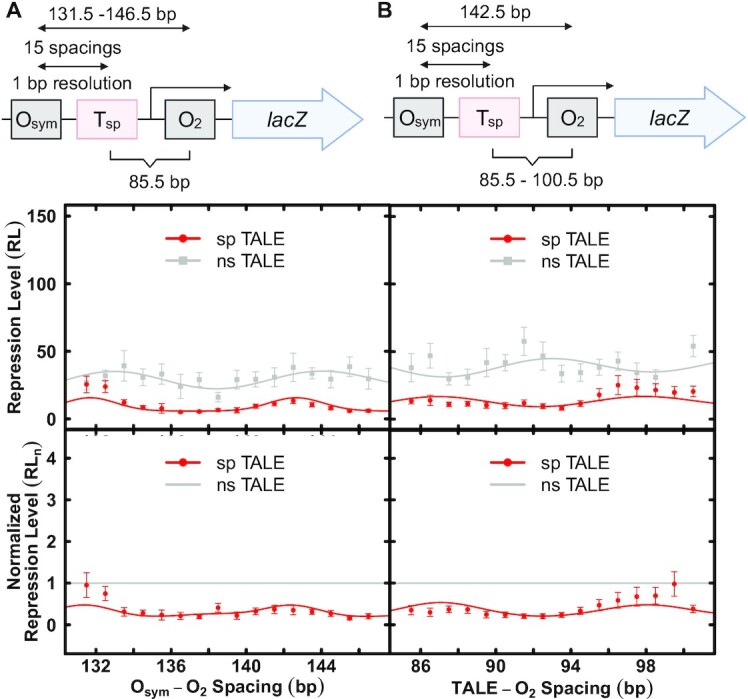
Effects of designed TALE proteins on Lac repressor-mediated DNA looping in living *E. coli*. (**A**) Series 1 construct. The top diagram indicates control element configuration. The upper graph depicts repression level (RL) as a function of center-to-center *lac* operator spacing in the presence of the indicated nonspecific (grey) or specific (red) TALE protein where the TALE site is at a fixed position relative to O_2_ in the presence of Lac repressor and 100 μM IPTG. The lower graph compares nonspecific or specific TALE effects when repression level values are normalized to the corresponding values for nonspecific designed architectural protein (grey line indicates a value of 1.0). (**B**) As in panel (A) except the data were obtained with Series 2 constructs illustrated in top diagram. All depicted curve fits are to the thermodynamic model described in methods, with fit parameters reported in Table 1.

We and others have engineered elements derived from the *lac* operon to create models allowing the study of DNA flexibility in living bacteria and effects of architectural DNA binding proteins on DNA looping (10,47,48). Intrigued by the idea of using designed sequence-specific TALE proteins as artificial architectural proteins to tune DNA looping, we began by designing reporter constructs (Figure 1F) containing the *lac* O_2_ operator in the proximal position to control *lacZ* transcription by RNA polymerase. Consistent with our past designs, an O_sym_ operator is then installed 131.5–146.5 bp (measured center-to-center) upstream from O_2_. The T_sp_ TALE recognition site is placed at a fixed position 85.5 bp upstream to the O_2_ operator (Series 1 constructs; Figure 1G; Figure 4A, top diagram; Supplementary Figure S1). Upon binding of the Lac repressor tetramer to both operators, a DNA loop is formed. Varying the distance between O_sym_ and O_2_ results in loops that differ in both length and twist strain. In a second set of experiments, the distance between O_sym_ and O_2_ operators was fixed at 142.5 bp (untwisted loop) and the T_sp_ TALE recognition site position was varied from 85.5 to 100.5 bp (measured center-to-center) upstream from the O_2_ operator (Series 2 constructs; Figure 1H; Figure 4B, top diagram; Supplementary Figure S2). This series rotates, at base-pair resolution, the placement of the artificial architectural DNA binding protein on different DNA faces within the DNA loop. In both Series 1 and Series 2 experiments, we hypothesized that the effects of artificial architectural proteins would depend on loop geometry and twist strain.

### The TALE as a DNA stiffening architectural protein to antagonize looping

TALEs were co-expressed with Series 1 and Series 2 reporter spacing constructs (Figure 4AB, top diagrams). Series 1 reporter constructs alter operator spacing in 1-bp increments to create repression loops of different lengths and twist strains. Repression is monitored by the repression level parameter (RL, Materials and Methods Equation (1)). It is important to emphasize that RL is defined by repression measurements obtained in the presence of sp TALE binding, so any effects of TALE binding on basal promoter function are taken into account by this parameter. In Series 1 reporter constructs (Figure 4A, upper graph) studied in the presence of ns TALE that cannot bind the loop, repression shows a modest dependence on operator spacing (grey data and fit) as expected for loops of this length, reflecting the expense of DNA twisting. Greatest repression occurs for untwisted loops with operator spacings near 133 and 144 bp, indicating an in vivo helical repeat of 11 bp/turn as commonly observed for negatively-supercoiled domains in bacteria. In striking contrast, sp TALE binding within the loop causes global derepression, consistent with the intended antagonism of looping by DNA stiffening (Figure 4A, upper graph, red data and fit). This effect is emphasized by normalizing to the data for the ns TALE (Figure 4A, lower graph).

Quantitative thermodynamic modeling data supporting these interpretations are presented in Table 1. The observed helical repeat (*hr*) is ∼11 bp/turn, setting the phasing for one periodic *sp*_optimal_ at ∼143 bp (∼13 helical turn operator separation). The fit values of the specific looping equilibrium constant, *K*_max_, in the presence of ns TALEs is 33.6. Importantly, this value decreases by a factor of ∼3 (9.8) for the sp TALE. *K*_max_ is a function of the maximal repression achievable, and thus the decrease in *K*_max_ seen with TALE binding simply reflects the inhibition of looping without information about the mechanism. This result parallels the observed decrease in RL values and shows that the sp TALE antagonizes DNA looping. The magnitude of the DNA twist constant, *C*_app_, reflects the difference between the maximal and minimal repression levels. Interestingly, values of the DNA twist constant, *C*_app_, in the presence of ns TALE is 0.44 but in the presence of the sp TALE the value becomes 2.86, exceeding the commonly accepted in vitro value of 2.40 (40). This striking result indicates that TALE binding creates a large obstacle to both DNA bending and twisting.

Thus, the results in Figure 4 and the corresponding fitting to the thermodynamic model are interesting in that they point to loop inhibition by TALE effects to limit both DNA bending and twisting. These effects are striking because TALE binding occupies only ∼15 bp of DNA, so effects on DNA both bending and twisting flexibility might be expected to be limited to only this segment of the DNA loop.

Similar results are obtained for Series 2 reporter constructs that incrementally reposition the T_sp_ TALE binding site within an untwisted repression loop where the *lac* operators are spaced by 142.5 bp (Figure 4B, top diagram). Comparing repression data obtained in the presence of a ns versus sp TALE, derepression is again observed for all positions with sp TALE binding, consistent with an intended DNA stiffening effect that should not depend on helical phasing (Figure 4B, upper graph). Again, normalization to repression data in the presence of the ns TALE emphasizes this result (Figure 4B, lower graph). Thus, global derepression is caused by sp TALE binding within the repression loop, consistent with a stiffening effect of sp TALE on DNA bending and twisting (Supplementary Tables S5 and S6). Quantitative data fitting to the thermodynamic model is again shown in Table 1, supporting these interpretations. In the presence of the ns TALE, the DNA twist constant, ^TALE^*C*_app_, is ∼0.25, ^TALE^*K*_Smax_ is ∼40 and ^TALE^sp_optimal_ is ∼93. A possible explanation for the observed phasing captured by ^TALE^*C*_app_ is that the TALE binding site may have some intrinsic curvature, and this sequence element is being systematically repositioning throughout a fixed loop. When compared to sp TALE, ^TALE^*C*_app_ increases to ∼0.34 and ^TALE^*K*_Smax_ decreases ∼2.6 fold to ∼16, again providing evidence that the designed TALE architectural protein stiffens DNA to both bending and twisting, antagonizing looping as hypothesized.

### The TALE-Nhp6A fusion as a DNA bending architectural protein to facilitate looping

**Figure 5.**
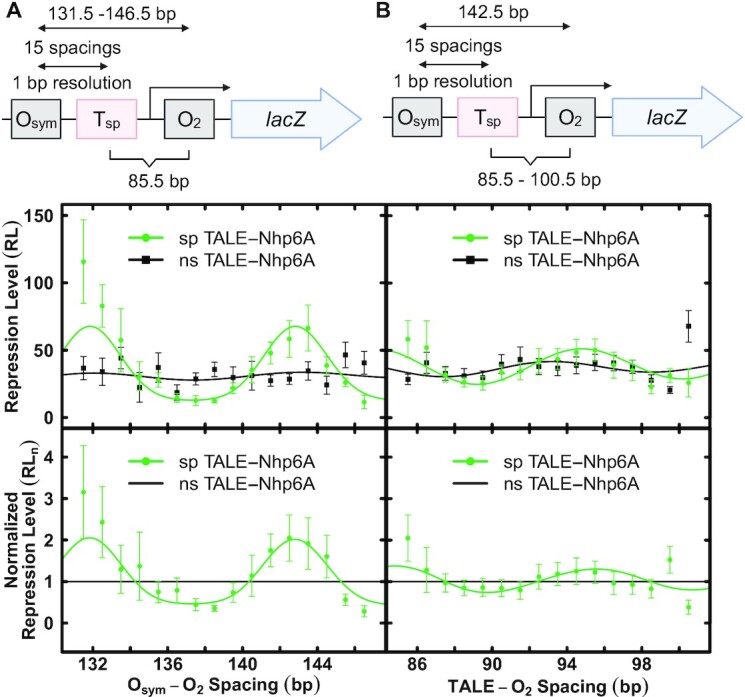
Effects of designed TALE-Nhp6A proteins on Lac repressor-mediated DNA looping in living *E. coli*. (**A**) Series 1 construct. The top diagram indicates control element configuration. The upper graph depicts repression level (RL) as a function of center-to-center *lac* operator spacing in the presence of the indicated nonspecific (black) or specific (green) TALE-Nhp6A protein where the TALE site is at a fixed position relative to O_2_ in the presence of Lac repressor and 100 μM IPTG. The lower graph compares nonspecific or specific TALE-Nhp6A effects when repression level values are normalized to the corresponding values for nonspecific designed architectural protein (black line indicates a value of 1.0). (**B**) As in panel (A) except the data were obtained with Series 2 constructs illustrated in top diagram. All depicted curve fits are to the thermodynamic model described in methods, with fit parameters reported in Table 1. Results of control experiments showing that lacI expression does not alter effective TALE protein concentrations and that TALE protein expression does not alter intrinsic lacI function are shown in Supplementary Figure S12.

We hypothesized that fusion with the sequence-nonspecific yeast Nhp6A HMGB DNA bending protein would allow targeted DNA bending within the repression loop to alter its energetics. Results are presented in Figure 5, showing an intriguing phasing dependence of TALE-Nhp6A on the DNA looping of Series 1 and Series 2 constructs. The repression level observed in the presence of the sp TALE-Nhp6A (Figure 5A, upper graph, green) is strongly altered relative to the data for the ns TALE-Nhp6A (Figure 5A, upper graph, black). Interestingly, sp TALE-Nhp6A strongly assists repression looping at operator spacings near 132 and 143 bp (about one helical turn apart) but inhibits looping at operator spacings near 137 and 148 bp (about one helical turn apart). Maxima and minima are thus separated by ∼ one half helical turn of DNA. The effect is emphasized when data are normalized to results in the presence of the ns TALE-Nhp6A protein (Figure 5A, lower graph, green). Thermodynamic model fitting is shown in Table 1. The value of *K*_max_ for the sp TALE-Nhp6A (∼55) is ∼2-fold larger than in the presence of the ns TALE-Nhp6A or ns TALE. This confirms that sp TALE-Nhp6A enhances DNA looping at relaxed loops, overcoming the inhibitory effect of sp TALE binding by a striking 5.6-fold. Interestingly, while the Nhp6A domain acts by decreasing DNA resistance to bending, fit values of *C*_app_ (Table 1) suggest that sp TALE-Nhp6A does not overcome the twist inhibition caused by sp TALE binding to T_sp_ (*C*_app_ only decreases to 2.11 from 2.86).

A phasing effect for TALE-Nhp6A binding is also observed in Series 2 reporter constructs where the TALE site is moved incrementally within a single untwisted repression loop (Figure 5B, top diagram). Although phasing of the unoccupied TALE binding sequence itself changes loop stability (Figure 5B, upper graph, black), the phasing effect is enhanced by sp TALE-Nhp6A binding. Optimal enhancement of the relaxed loop is observed at a T_sp_–O_2_ separation of ∼95 bp. Thus, twist-dependent loop stabilization and destabilization are induced by sp TALE-Nhp6A binding within the repression loop, consistent with a sequence-targeted bending effect of sp TALE-Nhp6A (Supplementary Tables S5 and S6). Quantitative data fitting supports this interpretation (Table 1). ^TALE^*K*_Smax_ increases ∼1.3-fold to 49.7 for sp TALE-Nhp6A (overall 3.2-fold change). ^TALE^*C*_app_ increases from ∼0.24 (ns TALE-Nhp6A) to ∼0.36 (sp TALE-Nhp6A). This again indicates that TALE binding inhibits DNA twisting, an effect that Nhp6A fails to rescue. It should be noted that ^TALE^*C*_app_ values are lower in Series 2 than *C*_app_ in Series 1 because the total loop size (142.5 bp) was deliberately chosen to be large (to accommodate multiple TALE positions) and near a looping probability maximum (relaxed loop).

### Modeling effects of artificial architectural proteins

To gain a better understanding of the observed effects of TALE and TALE-Nhp6A proteins on *lac* repression loop stabilization, we developed a series of molecular models based on a treatment of DNA at the level of base-pair steps with specific consideration of the spatial configuration of the bound repressor and designed architectural proteins (see Materials and Methods). For simplicity we ignored the large-scale opening of the Lac repressor detected in low-resolution structural studies (66,69–71) and consistent with fluorescence resonance energy transfer between dyes on designed, highly stable Lac repressor-mediated loops (72). We know from earlier work that opening of the Lac repressor alters the simulated configurations of certain loops but does not affect the predicted pattern of DNA loop formation significantly (73,74).

We first generated energy-optimized configurations of repressor-mediated, TALE-free loops to identify the helical repeat in protein-free DNA that best mimics the chain-length dependent repression profiles observed in the presence of ns TALE proteins. The looping propensities, that is, *J-*factors, extracted from the energies of TALE-free chains with a 10.9-bp/turn helical repeat exhibit local maxima at center-to-center operator spacings of 133.5 and 144.5 bp and a local minimum at a spacing of 138.5 bp (Supplementary Figure S7), values that match the pattern in the repression measurements in Figure 4A (series 1, grey data). The oscillations in the simulated profile reflect a mix of looped configurations dependent upon the operator spacing (see Figure 2D and 2E; Supplementary Figure S8; and the discussion below). The precise arrangement of the DNA operators against the repressor determines the lengths of duplex most likely to close into a loop. The energies are lowest and the looping most probable when the operators are in perfect register with the binding headpieces of the protein assembly, fit here to spatial arrangements in known high-resolution structures (54,55). The torsional stress, that is, the DNA under- and overtwisting, that builds up upon 5–6-bp changes in operator spacing results in a variety of high-energy configurations. These loops populate ‘transition states’ between energetically favored loops from different topological families. For example, the predicted enhancement in repression in TALE-less loops with 133.5 and 144.5 bp operator spacing derives from loops that are respectively under- and overtwisted at the intervening (138.5 bp) transition state. The peaks in looping propensity in one family of structures coincide with the valleys in the other and *vice versa*.

**Figure 6.**
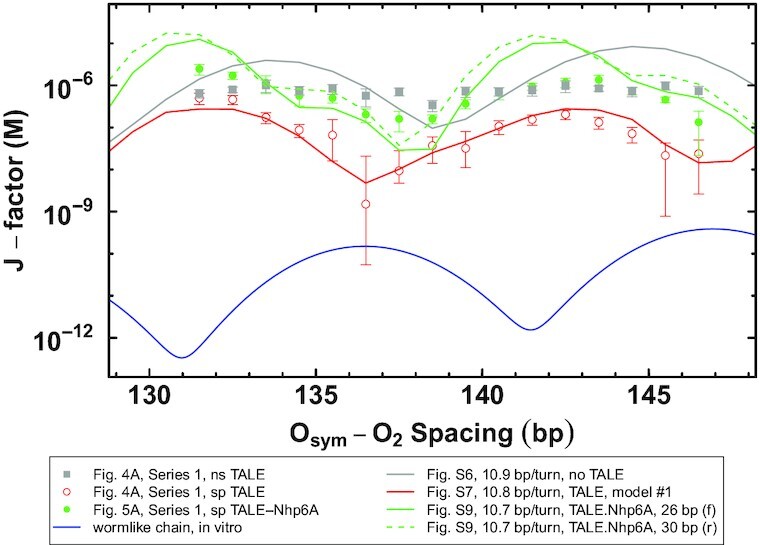
Comparison of experimental data with molecular modeling. Series 1 experimental data for nonspecific TALE (grey squares), specific TALE (open red circles), and specific TALE-Nhp6A (green circles) were transformed from the repression level data of Figures 4 and 5 into *J*-factor data. Included from the molecular models (lines) are *J*-factors of Lac repressor-mediated loops extracted from the energies of optimized loop configurations as a function of operator spacing. Profiles include TALE-free DNA with a 10.9-bp/turn DNA helical repeat (grey), TALE-bound DNA with protein-free DNA with a 10.8-bp helical repeat (red), and TALE-Nhp6A constructs with protein-free DNA with a 10.7-bp helical repeat (green) and either reversed or forward Nhp6A binding (dashed vs. solid green lines). A wormlike chain prediction of the in vitro *J*-factor (blue line) was generated with a torsional rigidity value, *C*, of 2.4 × 10^–19^ erg cm, a persistence length value, *P*, of 46.5 nm, and a helical repeat parameter, *hr*, of 10.48 bp/turn.

The predicted *J* factors of the protein-free loops tend to oscillate somewhat more widely and to exceed the maximum values extracted from the repression levels measured in the presence of ns TALE proteins (Figure 6, grey data). The discrepancies are, nevertheless, small and within the uncertainty of the protein-free DNA model. For example, the *J* factors of loops with 125.5–149.5-bp operator spacing and a 10.9-bp intrinsic helical repeat drop by ∼50% if the room-temperature fluctuations, that is, deformations that raise the energy by $\frac{1}{2}$ *k*_B_*T*, decrease from 4.8° to 4.7° (data not shown).

We next tested eight models of a TALE-bound construct derived from the high-resolution structure of the TAL effector PthXo1 in association with its DNA target sequence (16) to see which segments of the protein-DNA complex best capture the changes in amplitude and phasing of repression depicted in Figure 4A (series 1, red data). In the absence of a known structure of the 16 bp of DNA bound to the sp-TALE, the structures of eight overlapping 16-bp DNA segments from the PthXo1-DNA structure (16) were used as models of TALE binding (see Materials and Methods). While all of the models introduce a substantial reduction in the predicted ease of closing a DNA chain into a loop, only two exhibit a phase shift in the oscillatory variation of the *J*-factor with operator spacing compared to TALE-free DNA (Supplementary Figure S9, models 1 and 6). The two differ subtly from the others in terms of the constraints placed on the DNA loop; the distance between terminal base pairs is slightly smaller and the net bending between terminal base pairs, while small, occurs in a different direction. Moreover, the two representations of TALE-bound DNA yield looping profiles with maxima and minima closely matching the locations and magnitudes of the peaks and valleys in the repression data (Figure 6, red data) if the helical repeat of protein-free DNA is 10.8-bp/turn, with the change in intrinsic twist of protein-free DNA compensating for the ∼11.5-bp/turn DNA repeat within the TALE assembly. While the protein-bound element is held fixed in each simulation, the composite models provide a rough estimate of the range of local TALE-induced stiffening of the double helix and show the extent to which these limited motions impair the looping propensites of DNA (by an order of magnitude or more regardless of helical twist). The predicted looping propensity of TALE-bound DNA bearing model 1 is plotted along with the associated experimental data in Figure 6.

Finally we placed representative 10-bp fragments of Nhp6A-bound DNA on loops containing one of the selected TALE models. The fragments, taken from the collection of structures derived from NMR studies of the protein in complex with a 15-bp DNA duplex (29), were placed in two orientations and at variable locations downstream of the sp TALE construct on chains with a 10.7–10.8-bp helical repeat. Because the structure of the protein linker between the TALE and Nhp6A is unknown at high resolution, both dispositions of Nhp6A, and their similar impacts, are considered. The Nhp6A-induced bend and accompanying DNA undertwisting in the modeled pathways significantly enhance the looping propensities over those of TALE-free DNA in almost all cases (Supplementary Figure S10). The sites of highest looping propensity depend upon the spacing between the TALE and Nhp6A binding sites, with maxima consistent with the observed peaks in repression (Figure 5A, series 1, green data) occurring in constructs where the center of the Nhp6A-bound fragment lies 16 or 22 bp downstream of the center of the TALE-bound element and the protein-free segments have a 10.7-bp helical repeat. The enhancement pattern recurs but to a much lesser extent when Nhp6A is modeled an additional helical turn away, for example, 26 bp from the TALE site. The looping propensities of the TALE-Nhp6A-bearing chains are also lower than those of TALE-less loops at certain spacings, for example, in the vicinity of the minima when the Nhp6A lies 16 or 26 bp downstream of the TALE protein, in rough agreement with the repression data. Profiles with Nhp6A in the forward setting positioned 26 bp away from the TALE construct or 30 bp in the reverse setting are plotted along with the experimental data in Figure 6 (green data). Examining the experimental data, the apparent torsional rigidity for the loop with ns TALE is very low, is intermediate for specific TALE-Nhp6A, and is high (matching the in vitro expectation of the wormlike chain model) for the specific TALE. The differences in apparent DNA torsional rigidity in vivo and depending on different designed architectural proteins could be explained by a number of factors: (i) different degrees of participation of HU (or other nucleoid or architectural proteins) aiding twisting in vivo, (ii) different degrees of flexibility of Lac repressor protein (beyond the current treatment where the protein is modeled in a rigid ‘V’ conformation), (iii) supercoiled plectoneme geometry in vivo or (iv) non-wormlike chain behavior of the DNA.

The orientation of Nhp6A on DNA affects the site of protein uptake but does not change the character of the looping profiles. The predicted ease of looping is nearly identical for loops with Nhp6A bound in a reversed orientation 4 bp downstream of a site of forward binding (compare the profiles of loops with reversed versus forward Nhp6A uptake—dashed versus solid green lines—in Figure 6 and Supplementary Figure S10). Allowance for small 1–2 bp fluctuations in the downstream location of the Nhp6A relative to the TALE protein reduces the oscillations in the computed *J* factors, more closely capturing the magnitudes of values extracted from the repression data (Figure 6). The looping propensity is proportional to the net bending of the Nhp6A model, with the most highly bent pathways from the NMR ensemble increasing the *J* factors as much as order of magnitude over that depicted for 126.5–131.5-bp loops with 22-bp TALE-Nhp6A spacing and the most opened pathways decreasing the *J* factors by a factor of ∼5 (data not shown).

**Figure 7.**
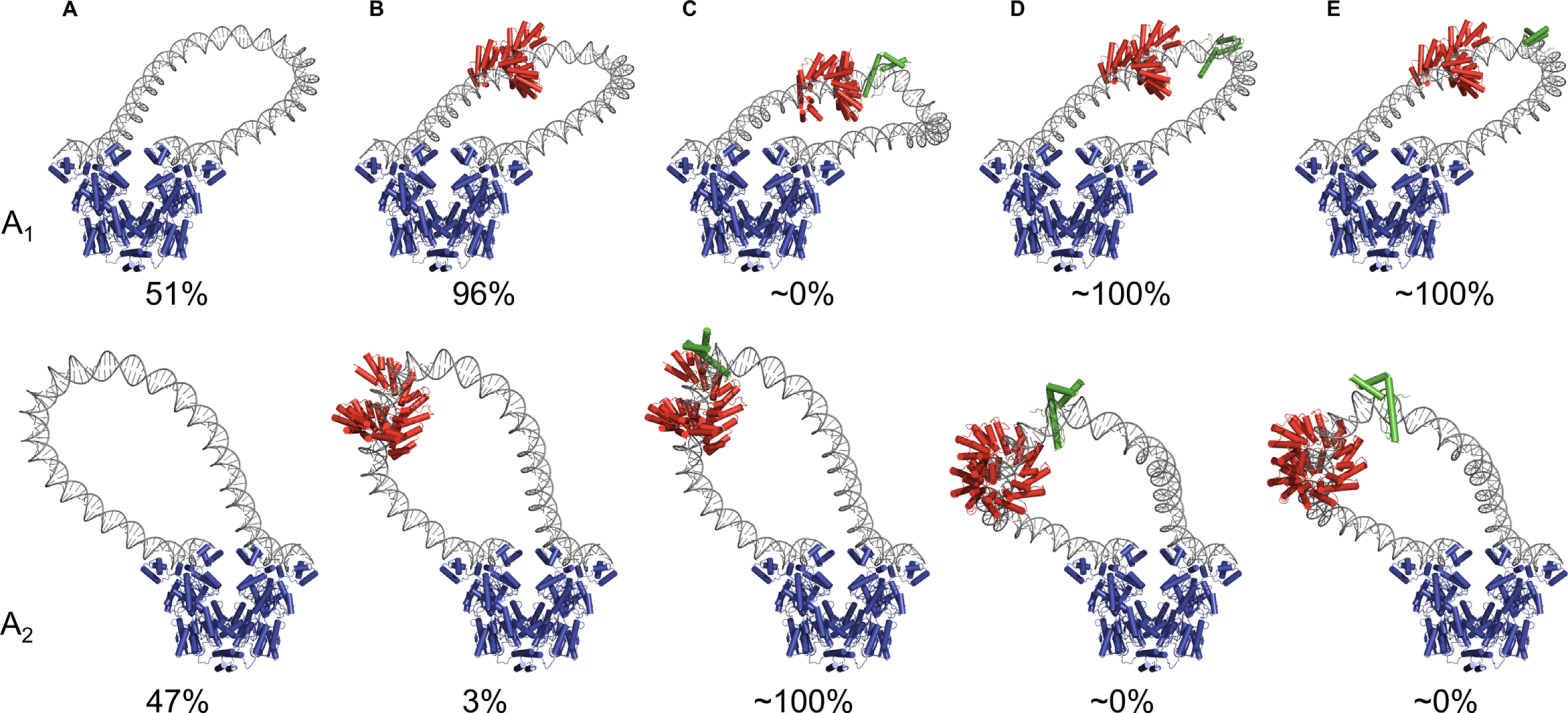
Models of DNA loop tuning by designed TALE-based architectural proteins. Energy-optimized configurations of Lac repressor-mediated DNA loops bearing TALE-Nhp6A constructs with O_sym_ and O_2_ operators spaced at distances of maximum looping propensity. The TALE construct is highlighted in red and the fused Nhp6A domain at predicted sites of likely binding in green. The repressor, depicted in blue, attaches to O_sym_ on its upper left edge and to O_2_ on its upper right edge. (**A**) TALE-less antiparallel loops with 133.5-bp spacing and a 10.9-bp intrinsic helical repeat on protein-free DNA. (**B**) TALE-associated loops with 131.5-bp spacing and a 10.8-bp repeat. (**C**-**E**) TALE-Nhp6A constructs with 131.5-bp spacing, a 10.7-bp repeat, and Nhp6A spaced respectively 16, 22 and 26 bp downstream of the TALE protein. The Nhp6A in C and D associates with DNA in a forward orientation and that in E in a reverse orientation. The effects of protein binding on the predicted proportions of looped states are shown as percentages below each image. Protein binding, however, has no effect on the structural family of these low-energy states (all from topological family F1). The pathway of the TALE-bound steps is represented by model 1 in Supplementary Table S8 and that of Nhp6A by model 4 from the ensemble of NMR-derived structures (pdb 1j5n) (29). See Supplementary Table S9 for the rigid-body step parameters and Supplementary Table S10 for the *J*-factors, structural families, and distances that must be spanned by the 65 amino acid residues separating the proline at the C terminus of the last repeat module of the TALE protein from the N-terminal methionine of Nhp6A in the dominant looped states.

Interestingly, the introduction of TALE-Nhp6A-bound fragments changes the predicted mix of configurational states adopted by the modeled DNA loops (Figure 7; Supplementary Figure S11). The loops formed most easily in the absence of architectural proteins, that is, with 133.5 and 144.5 bp center-to-center operator spacing, follow antiparallel pathways. The DNA enters and exits the repressor in opposing directions, forming relatively smooth turns through apices located closer to one of the ends of the loops than the other (Figure 7A). Whereas the loops labeled A_1_ depart the O_sym_ operator toward the center of the protein assembly—forming a turn closer to the 3′- than the 5′-end of the loop—the loops labeled A_2_ depart in the opposite direction, forming a turn closer to the 5′- than the 3′-terminus of the loop. The specific TALE binding site thus is accommodated along the long, relatively straight stretch of the TALE-free A_1_ loops and at or near the turn in the TALE-free A_2_ loops. Introduction of a stiff, straight fragment of TALE-associated DNA at the designated binding site thus stabilizes the A_1_ compared to the A_2_ configuration, not only increasing the energy of the A_2_ form compared to the A_1_ form but also increasing the energy of the protein-free segments in the TALE-associated loops over those in the TALE-less loops. The increase in total energy gives rise to both the reduced looping propensities of the protein-bound versus unbound loops and the difference in energy between the A_1_ and A_2_ configurations to the altered distribution of looped states (Figure 7B; Supplementary Figure S8). The specifically positioned TALE construct introduces more substantial distortions in the A_2_ than the A_1_ loops (see Supplementary Video S1).

The addition of Nhp6A to the TALE-bound models introduces further changes in the preferred looped states, stabilizing the A_2_ form significantly over the A_1_ form when Nhp6A is placed in a forward orientation 16 bp downstream of the TALE binding site (Figure 7C) and enhancing the A_1_ form over the A_2_ form when placed in the same orientation 22 bp downstream (Figure 7D; Supplementary Figure S8). In other words, the placement and orientation of Nhp6A, depicted in green in Figure 7, determine the balance between the looped states—a result reminiscent of the redistribution of loop populations previously found with different helical phasings of an intrinsically curved A-tract on slightly longer DNA chains (72). Looping configurations predicted when Nhp6A is bound in a reversed orientation are similar to those found when the protein is placed 4 bp closer to the TALE protein in a forward orientation (Figure 7E). TALE-Nhp6A binding is predicted to widen the mix of configurational states adopted by the least easily formed DNA loops. The protein-free loops are predicted to adopt a variety of high-energy pathways, including distorted forms of A_1_ and A_2_ looping from different structural families, as well as two loops, termed P_1_ and P_2_, with DNA entering and exiting the repressor in parallel directions. The P_1_ loop departs the O_sym_ operator in the same direction as the A_1_ loop, toward the central axis of the repressor, and the P_2_ loop in the same direction as the A_2_ loop, away from the repressor (see the variety of high-energy states in Supplementary Figure S11).

### Summary and implications

Beyond the intended goals of this study, our data, for the first time, demonstrate that TALE proteins strongly alter the bending and twisting properties of bound DNA, consistent with crowding of the DNA major groove by the TALE amino acids. Furthermore, our work gives insights into details of the mechanism of Nhp6A deformation of DNA. While tethered Nhp6A enhances DNA looping by inducing a targeted DNA kink, the direction of this deformation appears to be fixed and anisotropic, not creating a point of increased twist flexibility. These novel insights into the mechanical and dynamical properties of DNA bound by TALEs and/or Nhp6A would have been more difficult to observe and quantitate by other methods.

We believe that the combination of experimental gene repression data, thermodynamic modeling, and interpretation through biomechanical modeling of structure energetics, provides unusual insight into the behavior of *lac* repression loops modified by targeting of artificial architectural DNA binding proteins.

In summary, this work shows, for the first time, that sequence-specific DNA binding proteins based on TALEs and TALE fusions to the yeast Nhp6A protein can serve as targeted artificial architectural proteins that tune the probability of DNA looping in living bacteria by altering the mechanical properties of DNA.

## DATA AVAILABILITY

All salient data are included in the manuscript and supplemental information. Any other data are available from the authors on request.

## Supplementary Material

gkab759_Supplemental_Files
