# Supplementary material for: Designed architectural proteins that tune DNA looping in bacteria
Source: Nucleic Acids Res. 2021 Sep 3;49(18):10382–96. doi: 10.1093/nar/gkab759 (PMC8501960; doi:10.1093/nar/gkab759)
Supplement: gkab759_Supplemental_Files [file gkab759_supplemental_files.zip › Tse_2021_8.17_supplemental.pdf]

Supplemental material

Designed architectural proteins that tune DNA looping in bacteria

David Tse[1], Nicole A. Becker[1], Robert T. Young[2], Wilma K. Olson[2], Justin P. Peters[3],

Tanya L. Schwab[1], Karl J. Clark[1], and L. James Maher, III[1]*

[1]Department of Biochemistry and Molecular Biology
Mayo Clinic College of Medicine and Science
200 First St. SW
Rochester, MN 55905, USA

[2]Department of Chemistry and Chemical Biology
Rutgers, the State University of New Jersey
Center for Quantitative Biology
Piscataway, NJ 08854, USA

[3]Department of Chemistry and Biochemistry
University of Northern Iowa
1227 West 27th Street
Cedar Falls, IA 50614-0423

Table of contents

Table of contents	page
Supplemental Fig S1	3
Supplemental Fig S2	4
Supplemental Fig S3	5
Supplemental Fig S4	6
Supplemental Fig S5	7
Supplemental Fig S6	8
Supplemental Fig S7	9
Supplemental Fig S8	10
Supplemental Fig S9	11
Supplemental Fig S10	12
Supplemental Fig S11	13
Supplemental Fig S12	14
Supplemental Table S1	18
Supplemental Table S2	19
Supplemental Table S3	20
Supplemental Table S4	21
Supplemental Table S7	21
Supplemental Table S8	22
Supplemental Table S9	23
Supplemental Table S10	24

Supplemental Tables S5 & S6 are available within a separate excel file.
Supplemental Video S1 is available as a separate .mp4 file.

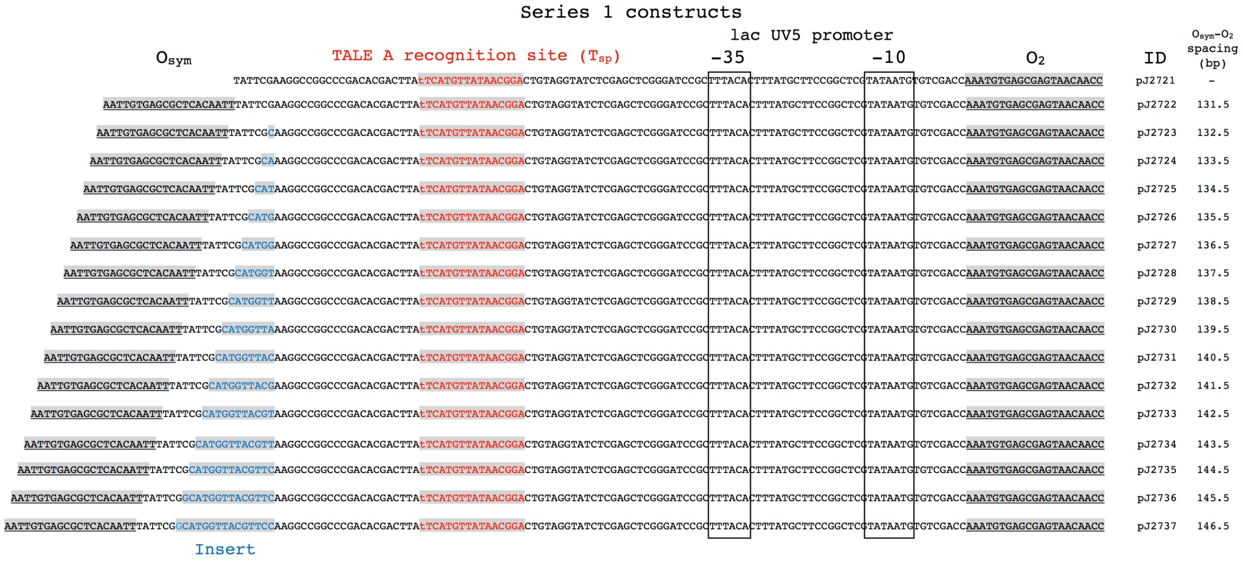

Supplemental Fig. S1. Sequences of control region elements of Series 1 reporter constructs. Key elements are shaded grey or boxed, as indicated. TALE binding sites, T_{sp}, are shown in red. Thymine residue recognized by the initial TALE invariant module is indicated in lower case. Operator spacings (center-to-center) are indicated for the shaded and underlined sequences.

Series 2 constructs

O_{sym} — TALE A recognition site (T_{sp}) — lac UV5 promoter (-35, -10) — O_2	ID	T_{sp}-O_2 spacing (bp)
AATTGTGAGCGCTCACAATTTATTCGGCATGGTCCTAGGGGCCGGCCCGACACGACTTAtTCATGTTATAACGGACTGTAGGTATCTCGAGCTCGGGATCCGCTTTACACTTTATGCTTCCGGCTCGTATAATGTGTCGACCAAATGTGAGCGAGTAACAACC	pJ2748	85.5
AATTGTGAGCGCTCACAATTTATTCGGCATGGTCCTAGGGGCCGCCCGACACGACTTAtTCATGTTATAACGGAGCTGTAGGTATCTCGAGCTCGGGATCCGCTTTACACTTTATGCTTCCGGCTCGTATAATGTGTCGACCAAATGTGAGCGAGTAACAACC	pJ2749	86.5
AATTGTGAGCGCTCACAATTTATTCGGCATGGTCCTAGGGGCCGCCGACACGACTTAtTCATGTTATAACGGAGCCTGTAGGTATCTCGAGCTCGGGATCCGCTTTACACTTTATGCTTCCGGCTCGTATAATGTGTCGACCAAATGTGAGCGAGTAACAACC	pJ2750	87.5
AATTGTGAGCGCTCACAATTTATTCGGCATGGTCCTAGGGGCCGCGACACGACTTAtTCATGTTATAACGGAGCCCTGTAGGTATCTCGAGCTCGGGATCCGCTTTACACTTTATGCTTCCGGCTCGTATAATGTGTCGACCAAATGTGAGCGAGTAACAACC	pJ2751	88.5
AATTGTGAGCGCTCACAATTTATTCGGCATGGTCCTAGGGGCCGGACACGACTTAtTCATGTTATAACGGAGCCCCTGTAGGTATCTCGAGCTCGGGATCCGCTTTACACTTTATGCTTCCGGCTCGTATAATGTGTCGACCAAATGTGAGCGAGTAACAACC	pJ2752	89.5
AATTGTGAGCGCTCACAATTTATTCGGCATGGTCCTAGGGGCCGACACGACTTAtTCATGTTATAACGGAGCCCGCTGTAGGTATCTCGAGCTCGGGATCCGCTTTACACTTTATGCTTCCGGCTCGTATAATGTGTCGACCAAATGTGAGCGAGTAACAACC	pJ2753	90.5
AATTGTGAGCGCTCACAATTTATTCGGCATGGTCCTAGGGGCCGCACGACTTAtTCATGTTATAACGGAGCCCGACTGTAGGTATCTCGAGCTCGGGATCCGCTTTACACTTTATGCTTCCGGCTCGTATAATGTGTCGACCAAATGTGAGCGAGTAACAACC	pJ2754	91.5
AATTGTGAGCGCTCACAATTTATTCGGCATGGTCCTAGGGGCCGACGACTTAtTCATGTTATAACGGAGCCCGACCTGTAGGTATCTCGAGCTCGGGATCCGCTTTACACTTTATGCTTCCGGCTCGTATAATGTGTCGACCAAATGTGAGCGAGTAACAACC	pJ2755	92.5
AATTGTGAGCGCTCACAATTTATTCGGCATGGTCCTAGGGGCCGCGACTTAtTCATGTTATAACGGAGCCCGACACTGTAGGTATCTCGAGCTCGGGATCCGCTTTACACTTTATGCTTCCGGCTCGTATAATGTGTCGACCAAATGTGAGCGAGTAACAACC	pJ2756	93.5
AATTGTGAGCGCTCACAATTTATTCGGCATGGTCCTAGGGGCCGGACTTAtTCATGTTATAACGGAGCCCGACACCTGTAGGTATCTCGAGCTCGGGATCCGCTTTACACTTTATGCTTCCGGCTCGTATAATGTGTCGACCAAATGTGAGCGAGTAACAACC	pJ2757	94.5
AATTGTGAGCGCTCACAATTTATTCGGCATGGTCCTAGGGGCCGACTTAtTCATGTTATAACGGAGCCCGACACGCTGTAGGTATCTCGAGCTCGGGATCCGCTTTACACTTTATGCTTCCGGCTCGTATAATGTGTCGACCAAATGTGAGCGAGTAACAACC	pJ2758	95.5
AATTGTGAGCGCTCACAATTTATTCGGCATGGTCCTAGGGGCCGCTTAtTCATGTTATAACGGAGCCCGACACGACTGTAGGTATCTCGAGCTCGGGATCCGCTTTACACTTTATGCTTCCGGCTCGTATAATGTGTCGACCAAATGTGAGCGAGTAACAACC	pJ2759	96.5
AATTGTGAGCGCTCACAATTTATTCGGCATGGTCCTAGGGGCCGTTAtTCATGTTATAACGGAGCCCGACACGACCTGTAGGTATCTCGAGCTCGGGATCCGCTTTACACTTTATGCTTCCGGCTCGTATAATGTGTCGACCAAATGTGAGCGAGTAACAACC	pJ2760	97.5
AATTGTGAGCGCTCACAATTTATTCGGCATGGTCCTAGGGGCCGTAtTCATGTTATAACGGAGCCCGACACGACTCTGTAGGTATCTCGAGCTCGGGATCCGCTTTACACTTTATGCTTCCGGCTCGTATAATGTGTCGACCAAATGTGAGCGAGTAACAACC	pJ2761	98.5
AATTGTGAGCGCTCACAATTTATTCGGCATGGTCCTAGGGGCCGAtTCATGTTATAACGGAGCCCGACACGACTTCTGTAGGTATCTCGAGCTCGGGATCCGCTTTACACTTTATGCTTCCGGCTCGTATAATGTGTCGACCAAATGTGAGCGAGTAACAACC	pJ2762	99.5
AATTGTGAGCGCTCACAATTTATTCGGCATGGTCCTAGGGGCCGtTCATGTTATAACGGAGCCCGACACGACTTACTGTAGGTATCTCGAGCTCGGGATCCGCTTTACACTTTATGCTTCCGGCTCGTATAATGTGTCGACCAAATGTGAGCGAGTAACAACC	pJ2763	100.5

Supplemental Fig. S2. Sequences of control region elements of Series 2 reporter constructs. Key elements are shaded grey or boxed, as indicated. TALE binding sites, T_{sp}, are shown in red. Thymine residue recognized by the initial TALE invariant module is indicated in lower case. O_2 - TALE spacings (center-to-center) are indicated for the shaded and underlined sequences.

A.

N-term   68.2 kDa (651 aa)

PKKKRKVSWKDASGWSRVDLRTLGYSQQQQEKIKPKVRSTVAQHHEALVGHGFTHAHIVALSQHPAALGTVAVTYQHII
TALPEATHEDIVGVGKQWSGARALEALLTDAGELRGPPLQ

LDTGQLVKIAKR-GGVTAMEAVHASRNALTGAPLN	0	**t**
PDPGPVVAIASNGGGKQALETVQRLLPVLCQDHG	1	**T**
LTPDQVVAIASHDGGKQALETVQRLLPVLCQDHG	2	**C**
LTPDQVVAIASNIGGKQALETVQRLLPVLCQDHG	3	**A**
LTPDQVVAIASNGGGKQALETVQRLLPVLCQDHG	4	**T**
LTPDQVVAIASNNGGKQALETVQRLLPVLCQDHG	5	**G**
LTPDQVVAIASNGGGKQALETVQRLLPVLCQDHG	6	**T**
LTPDQVVAIASNGGGKQALETVQRLLPVLCQDHG	7	**T**
LTPDQVVAIASNIGGKQALETVQRLLPVLCQDHG	8	**A**
LTPDQVVAIASNGGGKQALETVQRLLPVLCQDHG	9	**T**
LTPDQVVAIASNIGGKQALETVQRLLPVLCQDHG	10	**A**
LTPDQVVAIASNIGGKQALETVQRLLPVLCQDHG	11	**A**
LTPDQVVAIASHDGGKQALETVQRLLPVLCQDHG	12	**C**
LTPDQVVAIASNNGGKQALETVQRLLPVLCQDHG	13	**G**
LTPDQVVAIASNNGGKQALETVQRLLPVLCQDHG	14	**G**
LTPDQVVAIASNIGGKQALE	15	**A**

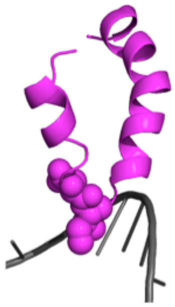

C-term   6.9 kDa (65 aa)

SIVAQLSRPDPALAALTNDHLVALACLGGRPAMDAVKKGLPHAPELIRRVNRRIGERTSHRVAS*

B.

N-term   68.2 kDa (651 aa)

PKKKRKVSWKDASGWSRVDLRTLGYSQQQQEKIKPKVRSTVAQHHEALVGHGFTHAHIVALSQHPAALGTVAVTYQHII
TALPEATHEDIVGVGKQWSGARALEALLTDAGELRGPPLQ

LDTGQLVKIAKR-GGVTAMEAVHASRNALTGAPLN	0	**t**
PDPGPVVAIASNGGGKQALETVQRLLPVLCQDHG	1	**T**
LTPDQVVAIASNIGGKQALETVQRLLPVLCQDHG	2	**A**
LTPDQVVAIASHDGGKQALETVQRLLPVLCQDHG	3	**C**
LTPDQVVAIASNIGGKQALETVQRLLPVLCQDHG	4	**A**
LTPDQVVAIASNIGGKQALETVQRLLPVLCQDHG	5	**A**
LTPDQVVAIASNNGGKQALETVQRLLPVLCQDHG	6	**G**
LTPDQVVAIASNGGGKQALETVQRLLPVLCQDHG	7	**T**
LTPDQVVAIASNNGGKQALETVQRLLPVLCQDHG	8	**G**
LTPDQVVAIASNNGGKQALETVQRLLPVLCQDHG	9	**G**
LTPDQVVAIASHDGGKQALETVQRLLPVLCQDHG	10	**C**
LTPDQVVAIASNGGGKQALETVQRLLPVLCQDHG	11	**T**
LTPDQVVAIASHDGGKQALETVQRLLPVLCQDHG	12	**C**
LTPDQVVAIASNIGGKQALETVQRLLPVLCQDHG	13	**A**
LTPDQVVAIASNGGGKQALETVQRLLPVLCQDHG	14	**T**
LTPDQVVAIASNGGGKQALE	15	**T**

C-term   6.9 kDa (65 aa)

SIVAQLSRPDPALAALTNDHLVALACLGGRPAMDAVKKGLPHAPELIRRVNRRIGERTSHRVAS*

C.

Nhp6A   10.8 kDa (93 aa)

MVTPREPKKRTTRKKKDPNAPKRALSAYMFFANENRDIVRSENPDITFGQVGKKLGEKWKALTPEEKQPYEAKAQADKK
RYESEKELYNATLA*

Supplemental Fig. S3: TALE and TALE-Nhp6A fusion protein sequences. A. The complete amino acid sequence of sp TALE (A) and ns TALE (B) are shown. The N- and C-terminal regions of both sp TALE and ns TALE are identical. The protein variability occurs in the 12th and 13th amino acid residues (red) of the 34-amino acid RVDs, compare target sequences (blue). A single 34-amino acid RVD (magenta cartoon), and its 12th and 13th amino acids (space-fill) are shown (A, right). (C) The 93-amino acid Nhp6A is included after the TALE C-terminal serine amino acid for constructs with Nhp6A.

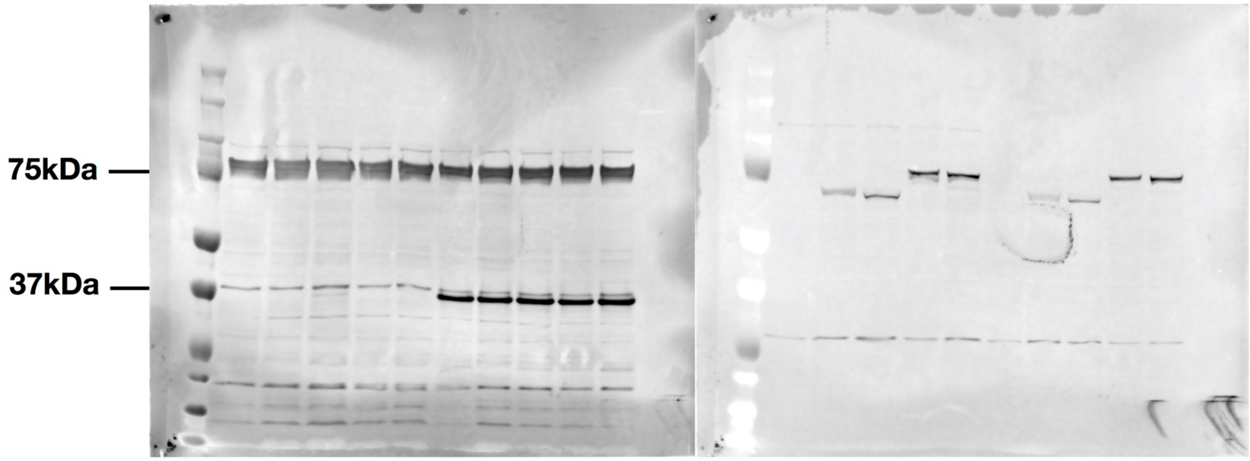

α-lacI α-V5

Supplemental Fig. S4. Uncropped images related to Fig. 3A.

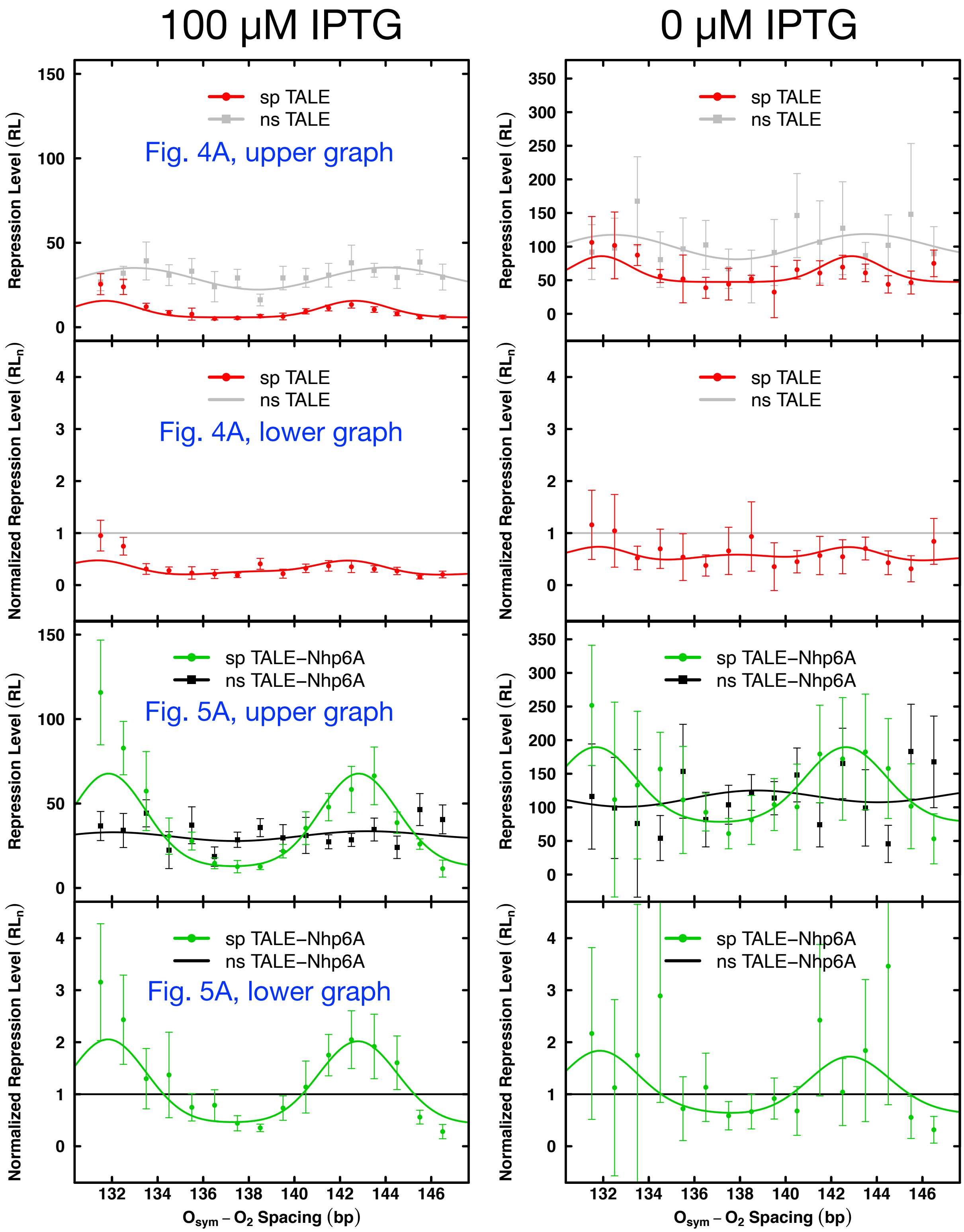

Supplemental Fig. S5. Experiments conducted in the absence of IPTG related to Series 1.

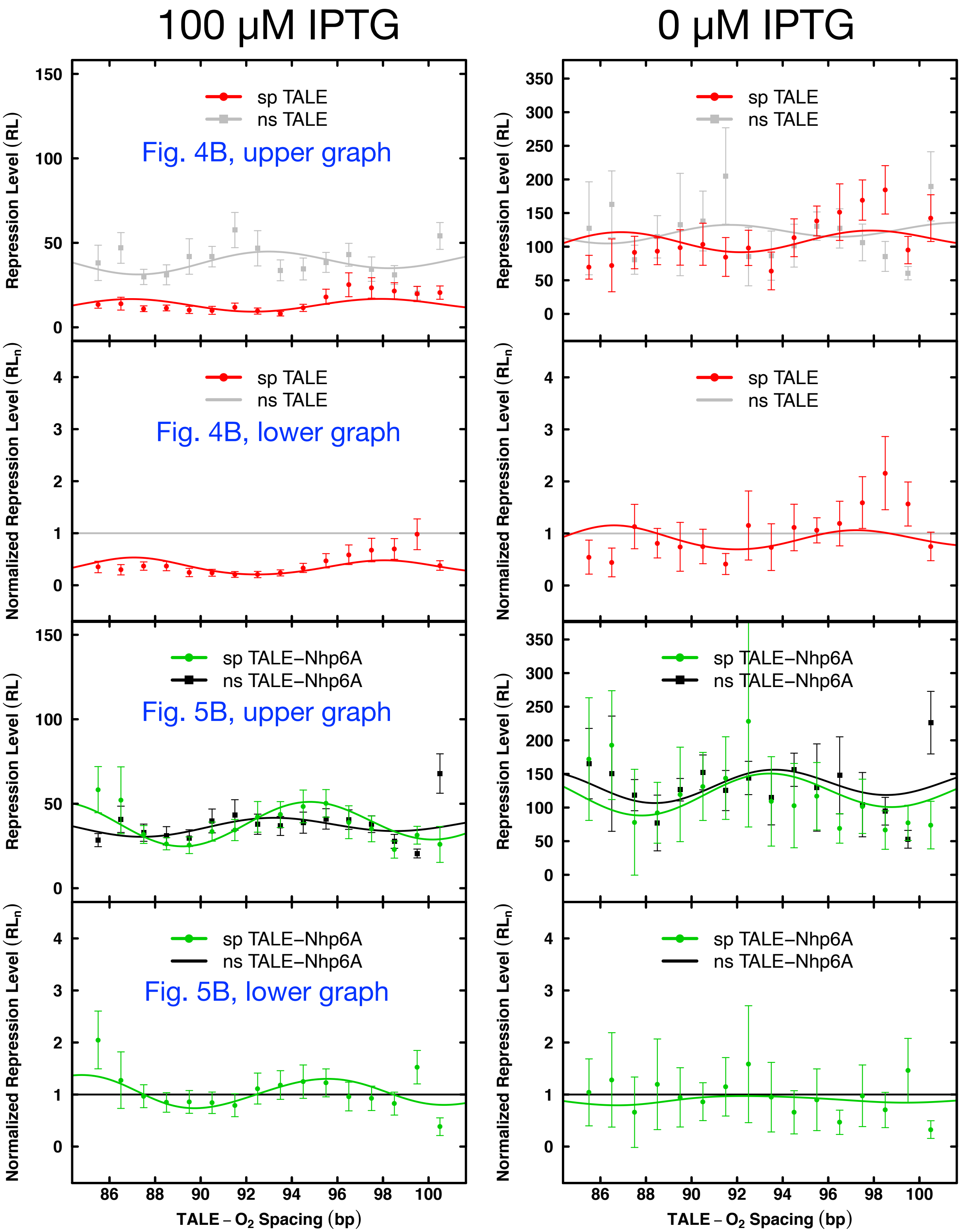

Supplemental Fig. S6. Experiments conducted in the absence of IPTG related to Series 2.

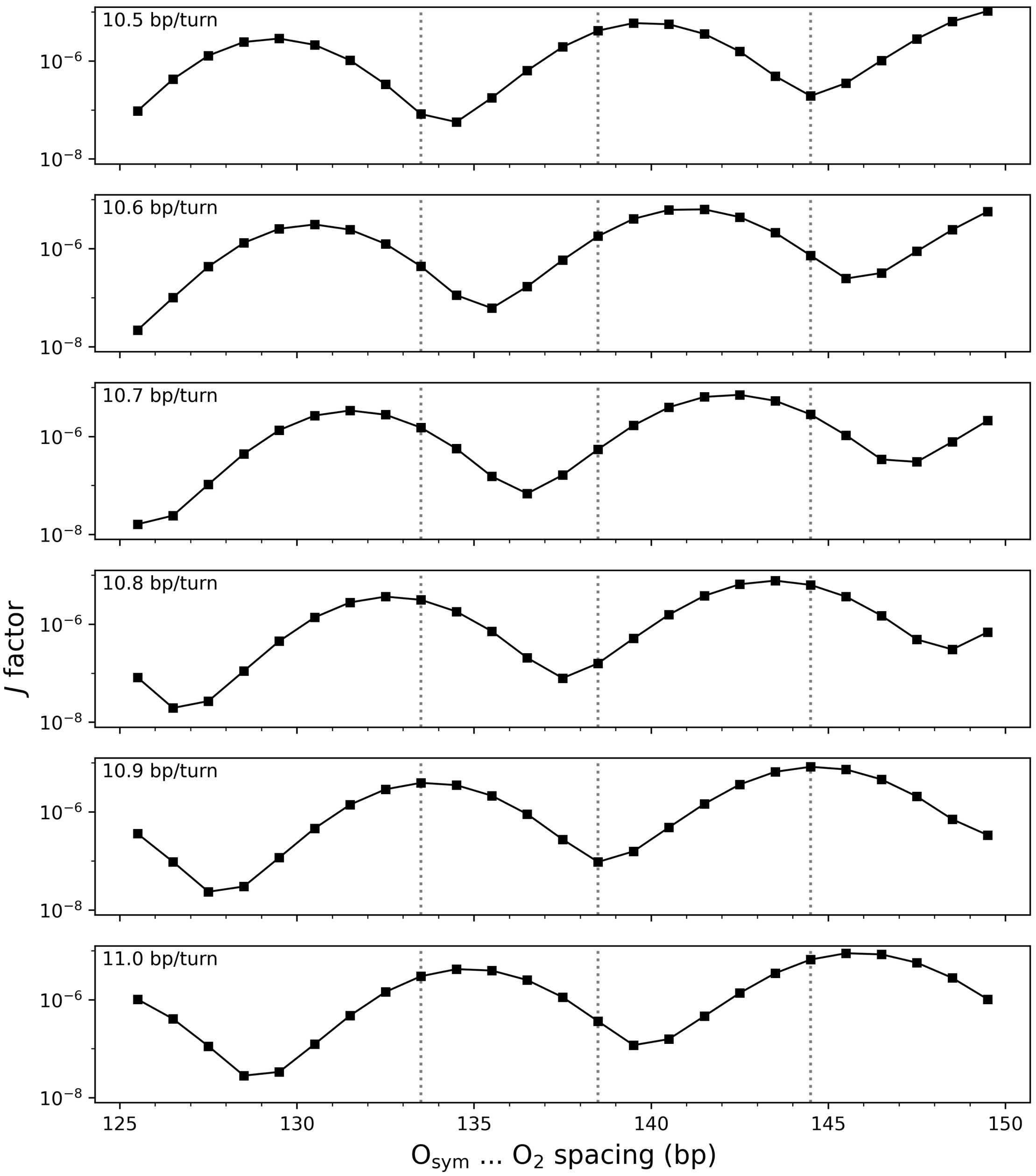

Supplemental Fig. S7. A 10.9-bp/turn DNA helical repeat yields looping profiles consistent with the chain-length dependent repression levels measured for TALE-free DNA. *J* factors of Lac repressor-mediated loops extracted from the energies of optimized loop configurations as a function of operator spacing. Protein-free DNA treated as an ideal, inextensible, naturally straight chain with elastic properties characteristic of mixed-sequence DNA and a 10.5 to 11 bp/turn helical repeat in its equilibrium rest state. The spacings associated with local maxima/minima in repression of TALE-less DNA (Fig. 4A: series I, grey data) are highlighted by dotted grey lines.

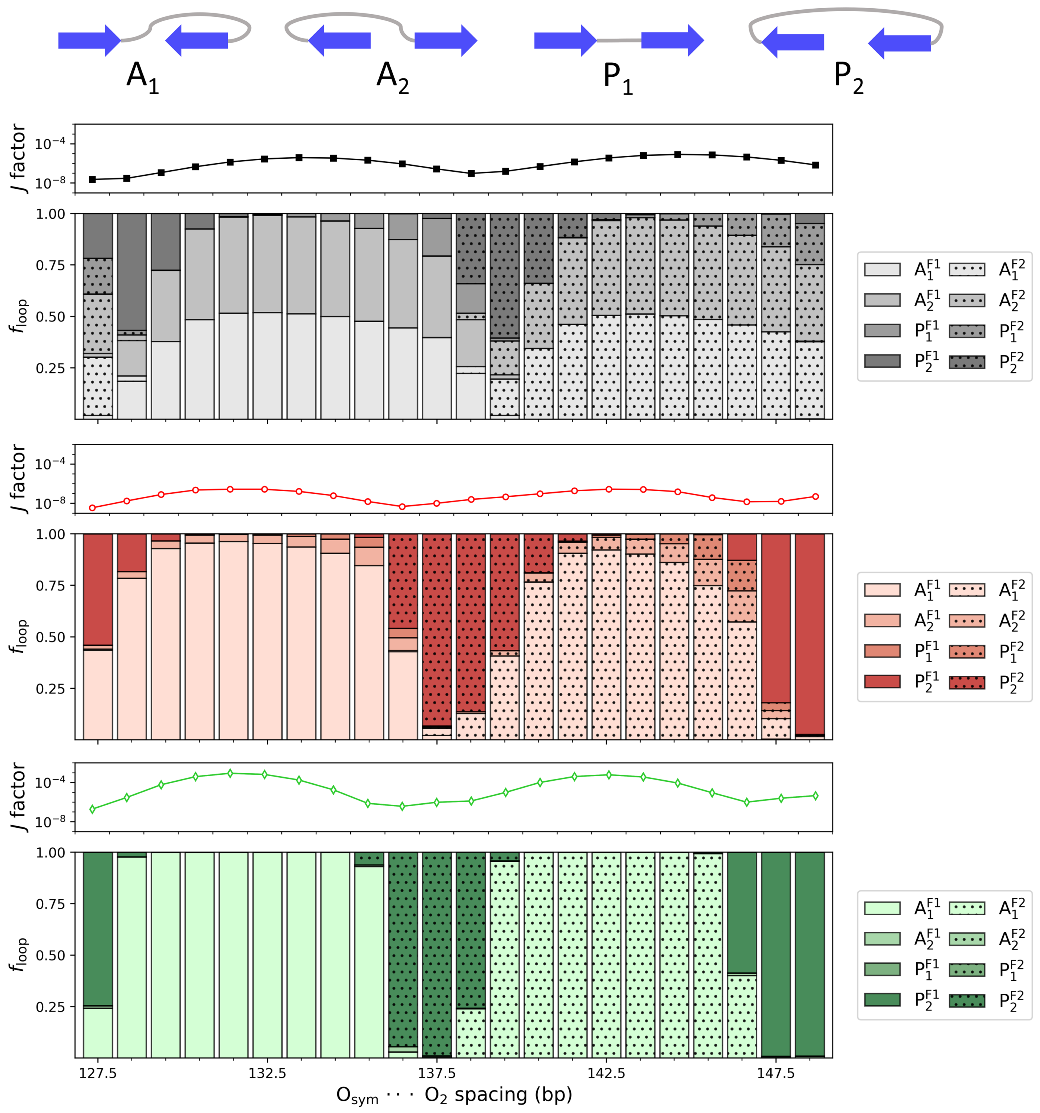

Supplemental Fig. S8. TALE and Nhp6A proteins alter the mix of configurations responsible for the predicted chain-length dependent profiles of DNA looping. Looping propensities J and fractional populations f_{loop} of Lac repressor-mediated loops extracted from the energies of optimized loop configurations in the absence of TALE proteins (grey line plots and histograms), with a TALE-bound element placed 85.5 bp upstream of the O2 operator (red line plots and histograms), and with a TALE-Nhp6A construct incorporating Nhp6A in a forward orientation 26 bp downstream of the TALE protein (green line plots and histograms). Note the transitions between structural families (open vs. patterned histograms) found in chains of low looping propensity and the redistribution of energetically favorable antiparallel configurations (relative proportions of the two most lightly shaded histograms) in the presence of bound protein. TALE-DNA binding described by model 1 and Nhp6A-DNA binding by model 4 from the ensemble of NMR-derived structures (1). Protein-free DNA assigned an intrinsic helical repeat of 10.9 bp in TALE-free loops, 10.8 bp in TALE-bound loops, and 10.7 bp in TALE-Nhp6A-bound loops. See the legend to Fig. 2 for details of the looping schematics (top row).

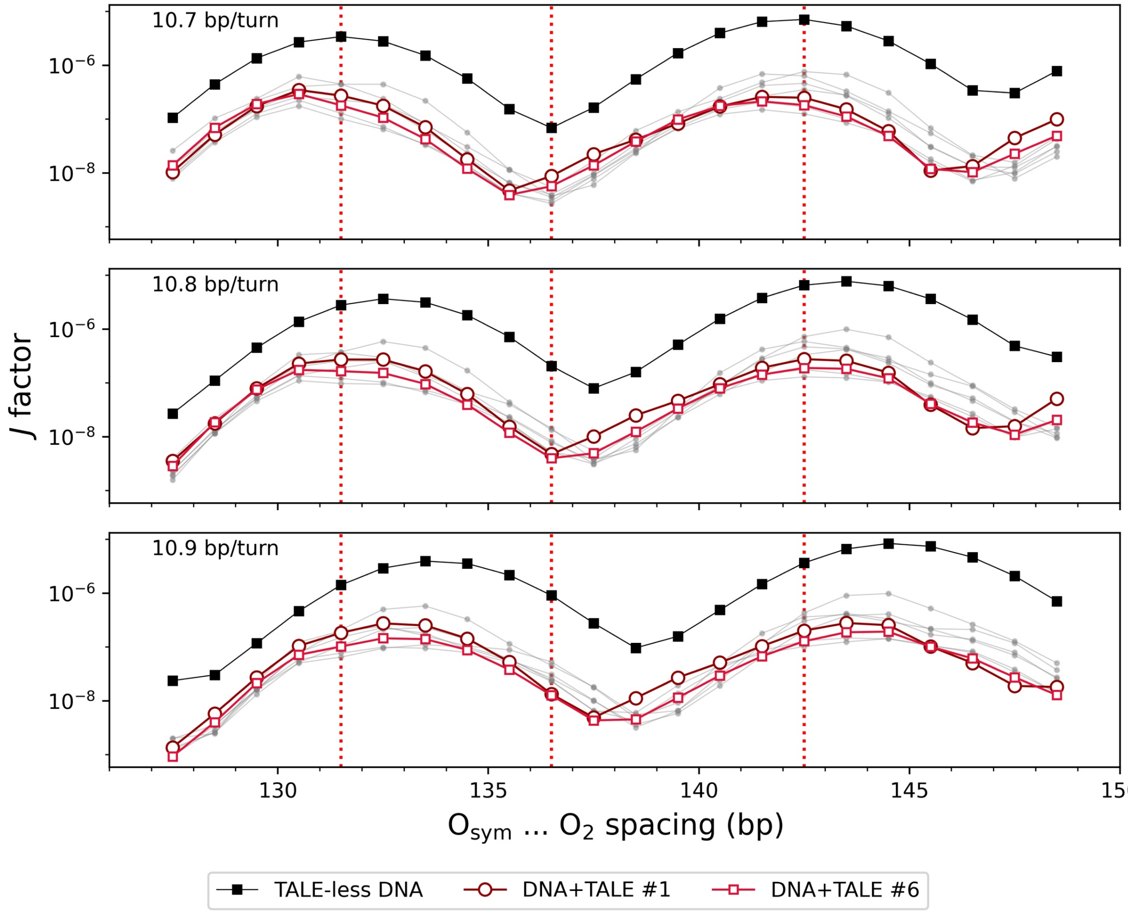

Supplemental Fig. S9. Selected 16-bp fragments from the complex of DNA with the TALE PthXo1 (2) introduce changes in DNA looping that mirror the changes in the amplitude and phasing of repression found in TALE-bound DNA. *J* factors of Lac repressor-mediated loops bearing TALE-bound elements extracted from the cited high-resolution structure and placed 85.5 bp upstream of the O2 operator in chains with 126.5-148.5 bp operator spacings and protein-free DNA with a 10.7-10.9-bp/turn helical repeat. See Supplemental Table S8 for the components of the high-resolution TALE-DNA complex incorporated in the modeled fragments. The looping profiles associated with the models 1 and 6, depicted respectively as large red circles and small red squares, show both a reduction in amplitude and a phase shift of the type shown in the repression levels of TALE-bound vs. TALE-less DNA. The models illustrated in light gray do not show a phase shift compared to TALE-less DNA (black squares). The spacing associated with local maxima/minima in repression of TALE-bound DNA (Fig. 4A: series I, red data) are highlighted by dotted red lines.

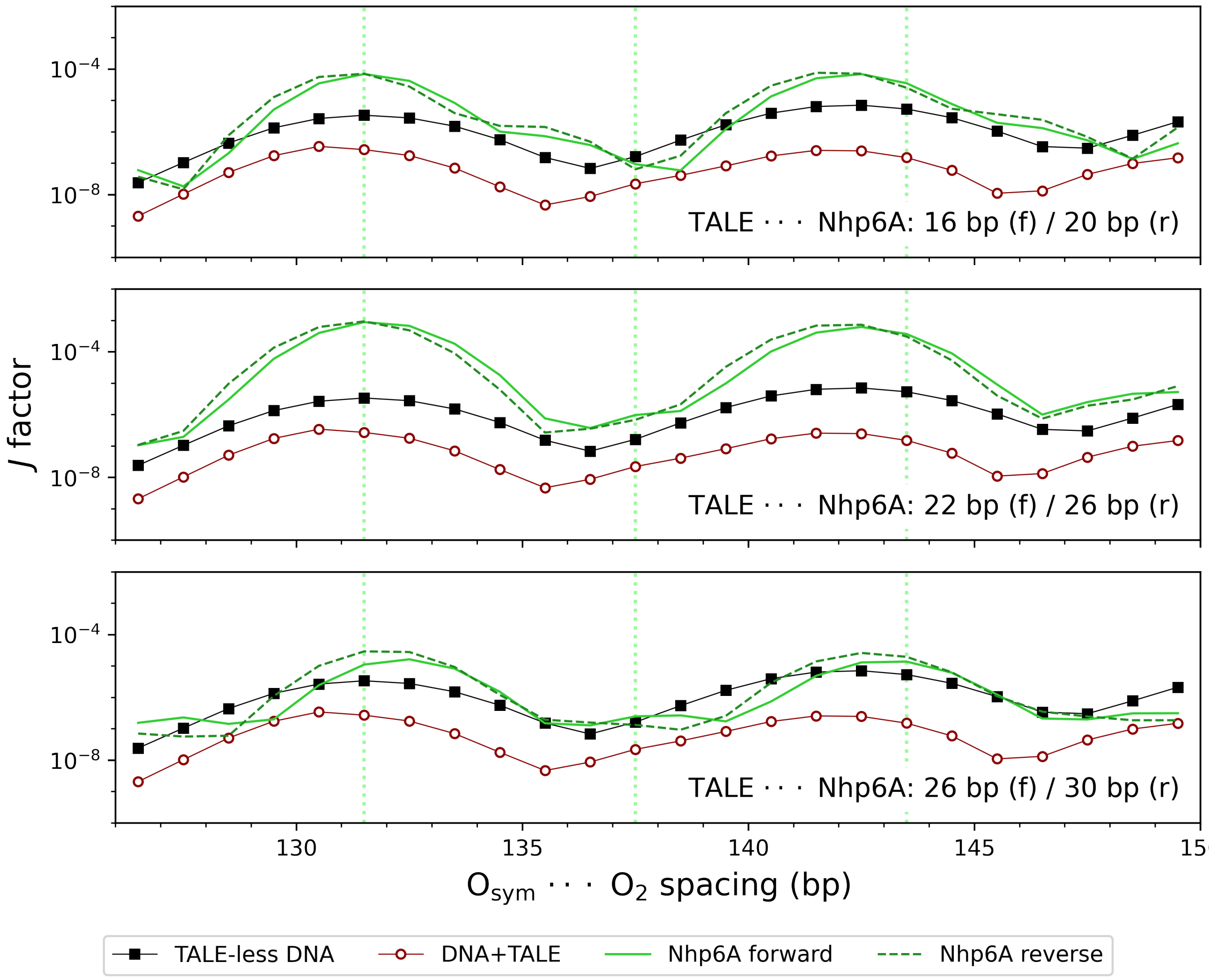

Supplemental Fig. S10. Selected settings of Nhp6A-bound DNA relative to the TALE construct on DNA introduce extremes of looping enhancement/depression of the type seen in the chain-length-dependent repression of loops bearing TALE-Nhp6A constructs. *J* factors of Lac repressor-mediated loops bearing TALE-Nhp6A constructs upstream of the O2 operator in chains with 126.5-148.5-bp operator spacings and protein-free DNA with a 10.7 bp helical repeat. The Nhp6A lies downstream of the TALE construct in forward and reverse settings (profiles depicted by solid/dashed green lines). The spacing between proteins is expressed in terms of the number of base pairs between the centers of TALE and Nhp6A uptake on DNA. The profiles of TALE-less (black squares) and TALE-bound (red circles) DNA are shown for comparison. The spacings associated with local maxima/minima in repression of TALE-Nhp6A-bound DNA (Fig. 5A: series I, green data) are highlighted by dotted green lines. Loops bearing TALE-bound elements described by model 6 are similar to those reported here using model 1 (see Supplemental Fig. S9). Chains modeled with a 10.8 bp helical repeat of protein-free DNA (data not shown) exhibit similar, albeit slightly out-of-phase, oscillatory looping profiles.

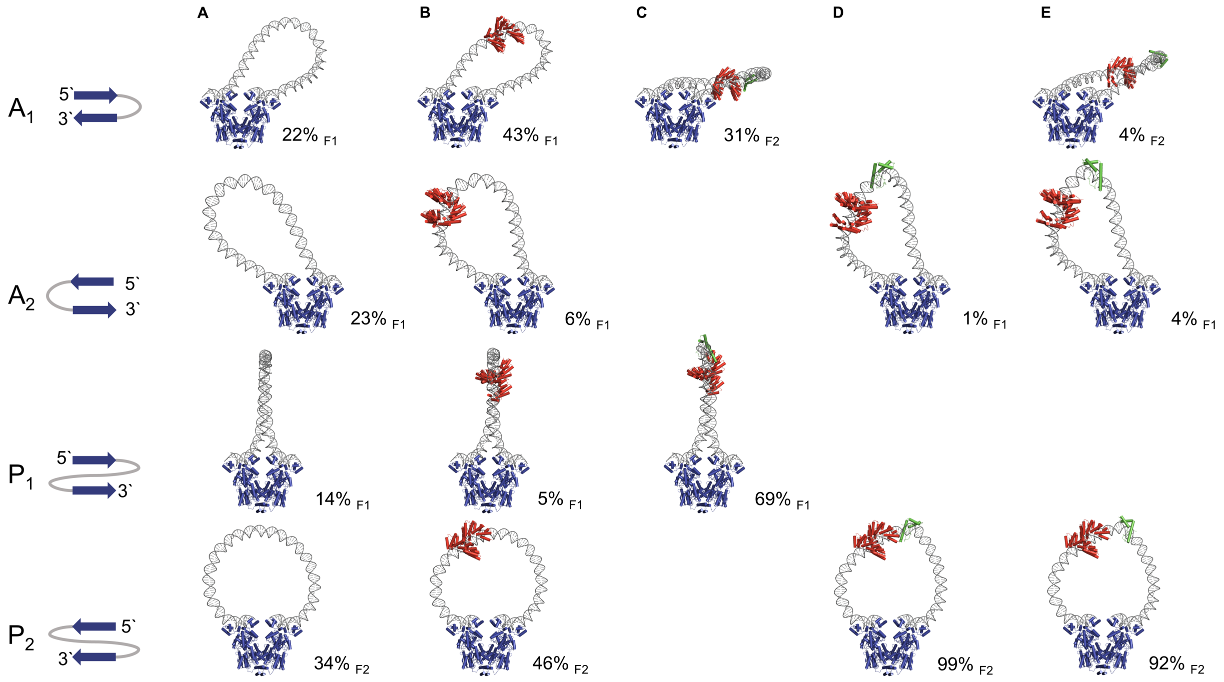

Supplemental Fig. S11. Designed TALE-based architectural proteins change the mix of configurational states found at points of minimum loop-closure propensity. The TALE construct is depicted in red and the fused Nhp6A domain in green. A. TALE-less antiparallel loops with 138.5-bp spacing and a 10.9-bp intrinsic helical repeat on protein-free DNA; B. TALE-associated loops with 136.5-bp spacing and a 10.8-bp repeat; C-E. TALE-Nhp6A constructs with 137.5-bp spacing, a 10.7-bp repeat, and Nhp6A incorporated respectively at sites 16, 22, and 26 bp downstream of the TALE protein. The Nhp6A in C and D associates with DNA in a forward orientation and that in E in a reverse orientation. Note the effects of protein uptake on the predicted proportions and configurational families of looped states (percentages and footnoted nomenclature to the right of each image). The pathway of the TALE-bound steps is represented by model 1 in Supplemental Table S8 and that of Nhp6A by model 4 from the ensemble of NMR-derived structures (pdb file 1j5n) (1). See Supplemental Table S10 for the *J* factors and distances that must be spanned by the 65 amino acid residues separating the C terminus of proline in the last repeat module of the TALE from the N-terminal methionine of Nhp6A in the dominant looped states. See the legend to Fig. 2 for details of the looping schematics in the left column.

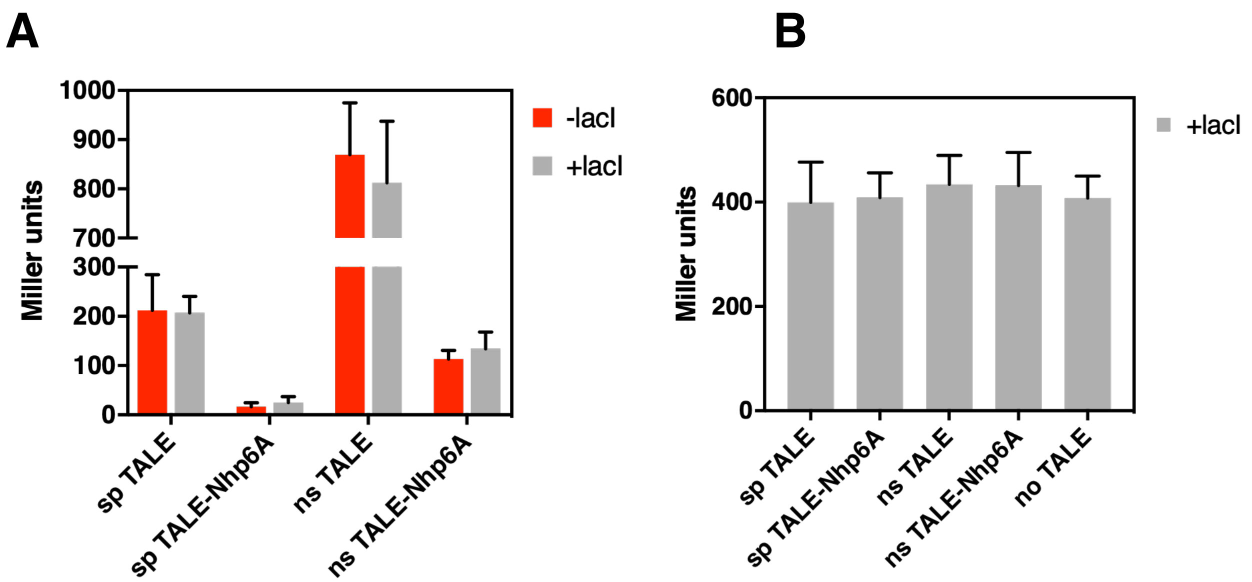

Supplemental Fig. S12. A. Reporter expression when the indicated TALE binding sites are placed in the promoter-proximal position and TALE proteins are expressed from the indicated source plasmids that do or do not contain a lacI expression casstte. Comparable levels of reporter repression indicate that lacI expression does not alter levels of TALE expression. B. Reporter expression when a single O2 operator is in the promoter-proximal position with no upstream operator and the indicated TALEs are co-expressed. Results indicate that TALE proteins do not affect lacI activity.

Supplemental Video S1.

Models of DNA loop tuning by designed TALE-based architectural proteins from different perspectives. This video, found in the accompanying mp4 file, highlights the configurational differences among Lac repressor-mediated loops bearing different TALE-Nhp6A constructs. The images convert from the conventional description of loop formation with respect to the V-shaped repressor assembly to different rotated viewpoints. Loops in A_1 and A_2 configurations progress from TALE-less to TALE-bound and TALE-Nh6pA-bound structures with 16, 22, and 26 bp between the centers of the TALE- and Nhp6A-bound sites. The table below gives the times when optimized structures with the specified molecular components appear in the video. See the legend to Fig. 7 for further details.

Video time index (sec)	DNA	TALE	Nhp6A 16 bp forward	Nhp6A 22 bp forward	Nhp6A 26 bp reverse
Conventional view showing V-shaped Lac repressor					
03:00	+				
04:00	+	+			
05:00	+	+	+		
06:00	+	+		+	
08:00	+	+			+
View in the direction of the operator axes following rotation about the vertical axis					
10:00	+				
12:00	+	+			
13:00	+	+	+		
15:00	+	+		+	
16:00	+	+			+
View into the V of the repressor following rotations about the horizontal and normal axes					
21:00	+				
23:00	+	+			
25:00	+	+	+		
26:00	+	+		+	
27:00	+	+			+

SUPPORTING INFORMATION METHODS

Treatment of protein-bound DNA

The Lac repressor assembly is represented by a rigid, V-shaped model, constructed by superposition of the structures of two well-resolved macromolecular complexes (3,4) —the 2.6-Å resolution X-ray crystallographic structure of the Lac protein dimer bound to the O_{sym} operator (PDB: 1efa) (5) and the 2.7-Å resolution X-ray structure of the tetrameric form of the protein without DNA-binding headpieces (PDB: 1lbi) (6). The DNA operators are assigned the rigid-body parameters adopted in the former complex, an approximation based on the similar spatial arrangements and comparable numbers of close protein-DNA contacts identified in NMR solution studies of the O_{sym} and O_2 operators bound to the N-terminal headpieces of the repressor protein (PDB: 1cjg and 2kej, respectively) (7,8). The overall deformation of the 15-bp modeled operators (see below), however, is somewhat greater than that deduced from the NMR data, introducing a greater bend between terminal base pairs compared to the corresponding stretches of O_{sym} and O_2 in the structural ensemble (70° in the current model vs. respective mean values of $41_{\pm 10}$° and $50_{\pm 6}$° in the NMR structures) but overwinding rather than underwinding (by +28.9° vs. –14.7° and –22.6° over the central 14-bp steps) relative to the same length of ideal B DNA. Here the uptake of twist is measured in terms of the twist of supercoiling (9,10), with the ideal chain assigned a twist of 360°/10.5 = 34.3° per base-pair step.

The model of the TALE construct is based on the configurations of protein-DNA fragments found within the 3.0-Å structure of the TALE PthXo1 from the rice pathogen *Xanthomonas oryzae* (PDB: 3ugm) in association with its naturally occurring DNA target sequence (2). The 36-bp duplex is broken into eight overlapping 16-bp fragments, each found using the SNAP component of the 3DNA/DSSR software (11,12) to have base-pair atoms in hydrogen-bonded contact with amino-acid atoms on the TAL effector (Supplemental Table S8) and each assigned the set of rigid-body parameters extracted from the corresponding DNA pathway. The Nhp6A-bound element is taken from the ensemble of 20 structures deduced from NMR studies of the complex of protein with a 15-bp DNA duplex containing a recognition sequence for the HMGB transcription factor SRY (PDB: 1j5n) (1). The protein-bound DNA is shortened to the ten base pairs found with SNAP (11,12) to be in direct hydrogen-bonded contact with atoms on protein, i.e., omitting the three base pairs at the 5′-ends and the two base pairs at the 3′-ends of the deposited structures. The Nhp6A-bound fragment is described, for simplicity, by two representative pathways (models 4 and 9 within the pdb file) with 47 and 48 of the 54 rigid-body parameters within one standard deviation of the average values at each step in the structural ensemble (Supplemental Table S9). Without prior knowledge of how and where the protein associates with the DNA loop, the Nhp6A-bound fragment is placed in two orientations at different locations with respect to the TALE-bound fragment. The protein is introduced so that the positively charged N-terminal tail of Nhp6A makes initial contact to DNA via either the leading strand or the complementary strand, here respectively termed forward vs. reverse orientation, with up to 30 bp separating the center of the Nhp6A-bound site from that of the TALE target site. Like the Lac repressor and the TAL effector, Nhp6A is treated as a ‘side group’ of DNA with the coordinates of amino-acid atoms expressed in the reference frame of one of the associated base pairs. Whereas the repressor slightly overwinds DNA, the TALE and Nhp6A proteins underwind DNA, with respective helical repeats of 10.4, 11.7, and 12.8 bp/turn (values obtained from the quotients of 360° and the average twists of supercoiling — 34.7°, 30.7°, and 28.2°).

The operators are aligned against the repressor such that the large protein-induced kink of DNA within each bound fragment lies on the CG base-pair step found to take up the distortion in the NMR structural ensembles (7,8). That is, whereas the central base-pair step of the modeled O_{sym} operator coincides with the highly kinked CG step at the center of the experimentally characterized system, the kink in the modeled O_2 operator lies off-center, on the CG step that incorporates the central G of the leading strand. The protein-bound DNA is restricted to the 15-bp steps in direct contact with repressor, with the three base pairs at the 3′-end of O_{sym} and the two base pairs at the 5′-end of O_2 treated as protein-free DNA. The three base pairs at the 5′-end of O_{sym} and the four base pairs at the 3′-end of O_2 are not included in the model.

Supplemental Table S1. TALE recognition sequences

TALE	recognition sequence
A (sp)	tTCATGTTATAACGGA
C (ns)	tTACAAGTGGCTCATT

Supplemental Table S2. Series 1 spacing construct plasmids used in this study

Plasmid	Distal Operator	Spacing (bp)*			Proximal Operator	T_{sp}
		$O_{sym}\cdots O_2$	$O_{sym}\cdots T_{sp}$	$T_{sp}\cdots O_2$		
pJ2644	-	-	-	-	T_A	-
pJ2646	-	-	-	-	T_C	-
pJ2721	-	-	-	85.5	O_2	+
pJ2722	O_{sym}	131.5	46	85.5	O_2	+
pJ2723	O_{sym}	132.5	47	85.5	O_2	+
pJ2724	O_{sym}	133.5	48	85.5	O_2	+
pJ2725	O_{sym}	134.5	49	85.5	O_2	+
pJ2726	O_{sym}	135.5	50	85.5	O_2	+
pJ2727	O_{sym}	136.5	51	85.5	O_2	+
pJ2728	O_{sym}	137.5	52	85.5	O_2	+
pJ2729	O_{sym}	138.5	53	85.5	O_2	+
pJ2730	O_{sym}	139.5	54	85.5	O_2	+
pJ2731	O_{sym}	140.5	55	85.5	O_2	+
pJ2732	O_{sym}	141.5	56	85.5	O_2	+
pJ2733	O_{sym}	142.5	57	85.5	O_2	+
pJ2734	O_{sym}	143.5	58	85.5	O_2	+
pJ2735	O_{sym}	144.5	59	85.5	O_2	+
pJ2736	O_{sym}	145.5	60	85.5	O_2	+
pJ2737	O_{sym}	146.5	61	85.5	O_2	+

*Denotes the base-pair distance (center to center) between the indicated elements

Supplemental Table S3. Series 2 spacing construct plasmids used in this study

Plasmid	Distal Operator	Spacing (bp)*			Proximal Operator	T_{sp}
		$O_{sym}\cdots O_2$	$O_{sym}\cdots T_{sp}$	$T_{sp}\cdots O_2$		
pJ2748	O_{sym}	142.5	57	85.5	O_2	+
pJ2749	O_{sym}	142.5	56	86.5	O_2	+
pJ2750	O_{sym}	142.5	55	87.5	O_2	+
pJ2751	O_{sym}	142.5	54	88.5	O_2	+
pJ2752	O_{sym}	142.5	53	89.5	O_2	+
pJ2753	O_{sym}	142.5	52	90.5	O_2	+
pJ2754	O_{sym}	142.5	51	91.5	O_2	+
pJ2755	O_{sym}	142.5	50	92.5	O_2	+
pJ2756	O_{sym}	142.5	49	93.5	O_2	+
pJ2757	O_{sym}	142.5	48	94.5	O_2	+
pJ2758	O_{sym}	142.5	47	95.5	O_2	+
pJ2759	O_{sym}	142.5	46	96.5	O_2	+
pJ2760	O_{sym}	142.5	45	97.5	O_2	+
pJ2761	O_{sym}	142.5	44	98.5	O_2	+
pJ2762	O_{sym}	142.5	43	99.5	O_2	+
pJ2763	O_{sym}	142.5	42	100.5	O_2	+

*Denotes the base-pair distance (center to center) between the indicated elements

Supplemental Table S4. TALE and TALE-Nhp6A plasmids used in this study

Plasmid	TALE	Nhp6A	LacI
pJ2657	-	-	-
pJ2662	-	-	+
pJ2658	A (sp)	-	-
pJ2663	A (sp)	-	+
pJ2660	A (sp)	+	-
pJ2665	A (sp)	+	+
pJ2741	C (ns)	-	-
pJ2745	C (ns)	-	+
pJ2743	C (ns)	+	-
pJ2747	C (ns)	+	+

Supplemental Tables S5 and S6 are available within a separate excel file.

Table S7. Thermodynamic model fits from Supplemental Figures S5 and S6

	O_{sym} – O_2 spacings			
Parameter	sp TALE	sp TALE-Nhp6A	ns TALE	ns TALE-Nhp6A
hr (bp/turn)	10.97 ± 0.01	10.97 ± 0.08	11.06 ± 0.51	11.12 ± 0.12
C_{app} (× 10^{-19} erg-cm)	3.49 ± 0.31	1.89 ± 0.15	0.40 ± 0.01	0.31 ± 0.01
K_{max}	38.40 ± 1.09	112.72 ± 1.46	113.57 ± 3.87	114.68 ± 4.19
K_{NSL}	46.43 ± 0.57	75.90 ± 1.99	0 ± 4.30	0 ± 5.04
$sp_{optimal}$ (bp)	142.89 ± 0.07	142.64 ± 0.02	143.49 ± 0.21	149.79 ± 0.12
	TALE – O_2 spacings			
${}^{TALE}C_{app}$ (× 10^{-19} erg-cm)	0.24 ± 0.01	0.30 ± 0.01	0.21 ± 0.01	0.26 ± 0.01
${}^{TALE}K_{Smax}$	116.32 ± 1.26	146.53 ± 1.35	121.67 ± 1.75	149.41 ± 1.47
${}^{TALE}sp_{optimal}$ (bp)	97.83 ± 0.10	93.41 ± 0.05	91.60 ± 0.13	93.61 ± 0.06

All parameters are +LacI in the absence of IPTG and are presented with a 95% confidence interval.

Supplemental Table S8. Protein-nucleic acid components of modeled TALE-DNA constructs.*

Model	Amino-acid chain/residues	Base-pair chain/residues	
	A	B	C
1	289-831	0-15	30-15
2	323-865	1-16	29-14
3	357-899	2-17	28-13
4	391-933	3-18	27-12
5	425-967	4-19	26-11
6	459-1001	5-20	25-10
7	493-1034	6-21	24-9
8	526-1068	7-22	23-8

*Chain identifiers and residue numbers of protein C^{α} and nucleic acid base atoms from pdb file 3ugm (2) incorporated in modeled 16-bp TALE-DNA constructs.

Supplemental Table S9. Rigid-body base-pair step parameters describing the average and specific closely related configurations of Nhp6A-bound DNA

Base-pair step	Rigid-body parameters					
Average*	⟨Tilt⟩, deg	⟨Roll⟩, deg	⟨Twist⟩, deg	⟨Shift⟩, Å	⟨Slide⟩, Å	⟨Rise⟩, Å
GG:CC	–0.9$_{\pm1.8}$	–16.2$_{\pm5.7}$	29.4$_{\pm1.6}$	–0.91$_{\pm0.32}$	–0.75$_{\pm0.33}$	3.09$_{\pm0.08}$
GT:AC	4.3$_{\pm3.5}$	–9.3$_{\pm8.8}$	43.9$_{\pm2.8}$	1.43$_{\pm0.54}$	1.32$_{\pm0.37}$	3.85$_{\pm0.39}$
TG:CA	–4.3$_{\pm3.2}$	2.6$_{\pm6.0}$	22.6$_{\pm5.2}$	–0.03$_{\pm0.13}$	–0.23$_{\pm0.26}$	3.08$_{\pm0.37}$
GA:TC	0.7$_{\pm2.0}$	10.1$_{\pm1.6}$	25.4$_{\pm2.1}$	–0.05$_{\pm0.12}$	–1.77$_{\pm0.15}$	3.46$_{\pm0.19}$
AT:AT	–1.2$_{\pm1.9}$	–7.3$_{\pm5.1}$	28.8$_{\pm3.5}$	0.01$_{\pm0.18}$	–0.74$_{\pm0.17}$	3.78$_{\pm0.35}$
TT:AA	–2.8$_{\pm1.9}$	24.8$_{\pm6.1}$	25.2$_{\pm2.6}$	–0.45$_{\pm0.17}$	–0.31$_{\pm0.23}$	3.30$_{\pm0.22}$
TG:CA	0.5$_{\pm1.3}$	3.9$_{\pm4.9}$	23.0$_{\pm2.1}$	–1.07$_{\pm0.17}$	–1.36$_{\pm0.26}$	3.23$_{\pm0.13}$
GT:AC	2.5$_{\pm0.8}$	18.7$_{\pm2.9}$	34.0$_{\pm1.0}$	0.69$_{\pm0.19}$	0.23$_{\pm0.07}$	3.80$_{\pm0.10}$
TT:AA	–1.5$_{\pm0.8}$	19.0$_{\pm1.3}$	40.4$_{\pm0.3}$	0.08$_{\pm0.06}$	0.06$_{\pm0.04}$	3.06$_{\pm0.09}$

Model 4†	ΔTilt, stdev	ΔRoll, stdev	ΔTwist, stdev	ΔShift, stdev	ΔSlide, stdev	ΔRise, stdev
GG:CC	0.23	0.16	0.53	0.09	0.65	0.13
GT:AC	0.67	2.14	0.31	0.32	0.45	0.20
TG:CA	0.75	1.48	0.35	0.01	0.04	0.27
GA:TC	1.59	0.59	0.54	0.38	0.82	1.14
AT:AT	0.03	0.64	0.40	0.29	0.13	0.05
TT:AA	0.13	0.09	1.02	0.11	0.67	0.59
TG:CA	0.05	0.73	0.44	0.94	0.21	0.58
GT:AC	0.11	0.40	1.09	0.71	0.70	0.45
TT:AA	0.26	1.66	0.52	0.24	0.26	0.74

Model 9†	ΔTilt, stdev	ΔRoll, stdev	ΔTwist, stdev	ΔShift, stdev	ΔSlide, stdev	ΔRise, stdev
GG:CC	0.41	0.08	0.87	0.08	0.77	0.19
GT:AC	0.59	2.00	0.19	0.29	0.87	0.34
TG:CA	0.94	1.77	0.08	0.52	0.09	0.64
GA:TC	1.87	1.08	0.84	0.02	0.38	1.36
AT:AT	0.09	0.62	0.29	0.78	0.35	0.09
TT:AA	0.38	0.09	0.30	0.33	0.31	0.32
TG:CA	0.06	0.94	0.38	1.04	0.49	0.02
GT:AC	0.02	0.04	0.54	0.66	0.90	0.19
TT:AA	0.32	0.04	0.65	0.46	0.46	0.38

*Values at base-pair steps in direct contact with protein averaged over the 20 structures included in pdb file 1j5n (1).

†Deviation of base-pair step parameters in the specified model expressed relative to the average value and standard deviation of each parameter at the given step, i.e., $\Delta\theta_{XX:YY} = (\theta_{XX:YY} - \langle\theta_{XX:YY}\rangle)/\sigma_{XX:YY}$, where $\theta_{XX:YY}$ is one of the six parameters at base-pair step XX:YY, $\langle\theta_{XX:YY}\rangle$ is the average value of the parameter over all reported structures, and $\sigma_{XX:YY}$ is the corresponding standard deviation (stdev).

Supplemental Table S10. Properties of modeled loops bearing TALE and Nhp6A proteins.*

$O_{sym}\cdots O_2$ spacing (bp)	TALE···Nh6pA spacing (bp)	Nh6pA orientation	Loop type	$J^{\dagger}$	$C^{\alpha}\cdots C^{\alpha\ddagger}$ (Å)
131.5	16	f	A_2^{F1}	7.0×10^{-5}	23.8
131.5	22	f	A_1^{F1}	8.9×10^{-4}	37.7
131.5	26	r	A_1^{F1}	9.4×10^{-4}	53.1
137.5	16	f	P_1^{F1}	6.5×10^{-8}	22.9
137.5	22	f	P_2^{F2}	9.7×10^{-7}	38.9
137.5	26	r	P_2^{F2}	6.3×10^{-7}	56.8

*Loops from Fig. 6 and Supplemental Fig. S7 with proportions greater than 10%
†Estimated *J* factors of loops of the specified types.
‡ Distances between the C^{α} atoms at the C terminus of proline in the last repeat module of the TALE protein to the N-terminal methionine of Nhp6A in the dominant configurations of the loops illustrated in Fig. 7 and Supplemental Fig. S11. Protein-free DNA modeled with a 10.7 bp helical repeat. Note the altered distances between chain ends upon re-orientation of Nhp6A.

Supplemental references

1. Masse, J.E., Wong, B., Yen, Y.M., Allain, F.H., Johnson, R.C. and Feigon, J. (2002) The S. cerevisiae architectural HMGB protein NHP6A complexed with DNA: DNA and protein conformational changes upon binding. *J Mol Biol*, **323**, 263-284.
2. Mak, A.N., Bradley, P., Cernadas, R.A., Bogdanove, A.J. and Stoddard, B.L. (2012) The crystal structure of TAL effector PthXo1 bound to its DNA target. *Science*, **335**, 716-719.
3. Swigon, D., Coleman, B.D. and Olson, W.K. (2006) Modeling the Lac repressor-operator assembly: the influence of DNA looping on Lac repressor conformation. *Proc Natl Acad Sci U S A*, **103**, 9879-9884.
4. Swigon, D. and Olson, W.K. (2008) Mesoscale modeling of multi-protein-DNA assemblies: the role of the catabolic activator protein in Lac-repressor-mediated looping. *Int J Non Linear Mech*, **43**, 1082-1093.
5. Bell, C.E. and Lewis, M. (2000) A closer view of the conformation of the Lac repressor bound to operator. *Nat Struct Biol*, **7**, 209-214.
6. Lewis, M., Chang, G., Horton, N.C., Kercher, M.A., Pace, H.C., Schumacher, M.A., Brennan, R.G. and Lu, P. (1996) Crystal structure of the lactose operon repressor and its complexes with DNA and inducer. *Science*, **271**, 1247-1254.
7. Spronk, C.A., Bonvin, A.M., Radha, P.K., Melacini, G., Boelens, R. and Kaptein, R. (1999) The solution structure of Lac repressor headpiece 62 complexed to a symmetrical lac operator. *Structure*, **7**, 1483-1492.
8. Romanuka, J., Folkers, G.E., Biris, N., Tishchenko, E., Wienk, H., Bonvin, A.M., Kaptein, R. and Boelens, R. (2009) Specificity and affinity of Lac repressor for the auxiliary operators O2 and O3 are explained by the structures of their protein-DNA complexes. *J Mol Biol*, **390**, 478-489.
9. Britton, L.A., Olson, W.K. and Tobias, I. (2009) Two perspectives on the twist of DNA. *J Chem Phys*, **131**, 245101.
10. Clauvelin, N., Olson, W.K. and Tobias, I. (2012) Characterization of the geometry and topology of DNA pictured as a discrete collection of atoms. *J Chem Theory Comput*, **8**, 1092-1107.
11. Lu, X.J. and Olson, W.K. (2008) 3DNA: a versatile, integrated software system for the analysis, rebuilding and visualization of three-dimensional nucleic-acid structures. *Nat Protoc*, **3**, 1213-1227.
12. Lu, X.J., Bussemaker, H.J. and Olson, W.K. (2015) DSSR: an integrated software tool for dissecting the spatial structure of RNA. *Nucleic Acids Res*, **43**, e142.
